# Fungal Fight Club: phylogeny and growth rate predict competitive outcomes among ectomycorrhizal fungi

**DOI:** 10.1093/femsec/fiad108

**Published:** 2023-09-11

**Authors:** Alexander H Smith, Laura M Bogar, Holly V Moeller

**Affiliations:** Department of Integrative Biology, University of Colorado, Denver Auraria Campus Science Building 1150 12th St, Denver CO 80204, USA; Department of Plant Biology, University of California, Davis, 605 Hutchison Dr Green Hall rm 1002 Davis CA 95616-5720, USA; Department of Ecology, Evolution and Marine Biology, University of California, Santa Barbara CA 93106-9620, USA

**Keywords:** co-culture experiment, competitive hierarchy, facilitation, mixed effects modeling, niche partitioning, pH tolerance

## Abstract

Ectomycorrhizal fungi are among the most prevalent fungal partners of plants and can constitute up to one-third of forest microbial biomass. As mutualistic partners that supply nutrients, water, and pathogen defense, these fungi impact host plant health and biogeochemical cycling. Ectomycorrhizal fungi are also extremely diverse, and the community of fungal partners on a single plant host can consist of dozens of individuals. However, the factors that govern competition and coexistence within these communities are still poorly understood. In this study, we used in vitro competitive assays between five ectomycorrhizal fungal strains to examine how competition and pH affect fungal growth. We also tested the ability of evolutionary history to predict the outcomes of fungal competition. We found that the effects of pH and competition on fungal performance varied extensively, with changes in growth media pH sometimes reversing competitive outcomes. Furthermore, when comparing the use of phylogenetic distance and growth rate in predicting competitive outcomes, we found that both methods worked equally well. Our study further highlights the complexity of ectomycorrhizal fungal competition and the importance of considering phylogenetic distance, ecologically relevant traits, and environmental conditions in predicting the outcomes of these interactions.

## Teaser

While interactions between ectomycorrhizal fungi are complex, considering ecologically relevant traits, phylogenetic relatedness, and environmental conditions all together may provide insight into competitive outcomes.

## Introduction

Ectomycorrhizal fungi (EMF) are obligate plant mutualists that associate with 60% of all tree stems on Earth (Steidinger et al. 2019) and participate in nutrient trading that is especially important for woody temperate plants (Smith and Read 2008). These fungi are important even in the early stages of a host plant’s life, as seedling survival and biomass are directly correlated with a host’s ability to acquire fungal partners (Onguene and Kuyper 2002). Benefits of the mycorrhizal symbiosis for host plants include the physical extension of a host’s resource pool, utilization of enzymes to access recalcitrant nutrients, and even protection against root pathogens (Leake et al. 2004). Because of these functions, EMF play a key role in forest carbon and nitrogen cycling and above- and belowground diversity (Leake et al. 2004). Additionally, EMF are both taxonomically and functionally diverse. For example, EMF vary in enzymatic activity (Courty et al. 2005), mycelial growth (Cairney 1999), and host preference (Tedersoo et al. 2008). Thus, fungal community composition can affect both large-scale ecological cycles as well as the health of an individual host plant. Understanding how these communities assemble as a function of biotic and abiotic interactions is therefore paramount to the field of forest ecology.

Competition is a key factor structuring communities of EMF (Koide et al. 2005, Kennedy 2010). Ectomycorrhizal fungal communities are complex mosaics, even on the scale of meters (Taylor and Bruns 1999, Zhou and Hogetsu 2002, Anderson et al. 2014), and a single ectomycorrhizal plant host can harbor tens to hundreds of EMF in its rhizosphere (Bahram et al. 2011, Thoen et al. 2019), even multiple genets of the same fungal species (Hortal et al. 2012). This diversity within small spatial scales would suggest that EMF may tend to occupy similar substrates or use overlapping soil resources. Consistent with this, many EM fungal communities appear to be structured by competition (Wu et al. 1999, Koide et al. 2005, Pickles et al. 2012). Even their response to environmental factors like temperature and their relative investment in symbiotic and non-symbiotic tissues can be affected by the presence of a competitor (Hortal et al. 2016). Furthermore, different species of EMF differ in both competitive ability (Kennedy et al. 2007, Maynard et al. 2017) and mutualistic function (i.e. decomposition ability and enzymatic activity) (Lindahl and Tunlid 2015, Moeller and Peay 2016), and it has been shown that host plant performance can be impaired by associating with several competing fungi (Kennedy et al. 2007). Being able to predict the outcomes of competition between species will have implications both for fungal ecology and for the health of host plants (Kennedy et al. 2007, Hortal et al. 2017).

In addition to competition, abiotic factors can also structure these communities. Properties such as soil pH (Yamanaka 2003, Gryndler et al. 2017, Davison et al. 2021), temperature (Davison et al. 2021), and nutrient availability (Huggins et al. 2014, Sterkenburg et al. 2015) have all been observed to change the composition of mycorrhizal fungal communities. In particular, pH can affect fungal growth in vitro (Hung and Trappe 1983, Yamanaka 2003) and in situ (Ge et al. 2017, Glassman et al. 2017). Possible mechanisms by which pH affects fungal performance include changes in enzymatic efficacy (Leake and Read 1990) and spore production and germination (Siqueira et al. 1984, Coughlan et al. 2000). pH also has been shown to vary in orders of magnitude across small spatial and temporal scales (Blossfeld et al. 2013), affecting microbial communities (Lauber et al. 2009). Thus, an EM fungus may have to adapt to different pH environments, both across its own mycelium and as it disperses to new environments. The extent to which an individual EM fungus succeeds in adjusting to these variations in pH may have powerful effects on competitive outcomes and community structure in soils.

While it is important to understand how competition and abiotic factors individually affect ectomycorrhizal fungal community structure, competitive interactions and community assembly happen in the context of the abiotic environment, and so, can be affected by environmental factors. For example, Mujic et al. (2016) demonstrated in a greenhouse study that abiotic soil conditions can change the outcomes of competitive interactions between fungi, with heterogeneous soil facilitating coexistence. Likewise, the competitive dominance of two EMF species (*Piloderma*) reversed with the addition of wood ash to the growth substrate (Mahmood 2003). These studies made complex alterations to substrate chemistry, but there is also evidence that pH specifically is an important factor affecting competition between fungi. For example, it has been shown in yeast that competitive ability, through secreted toxins, has a narrow optimal pH window, suggesting that yeast community composition is controlled by environmental pH (Chen and Chou 2017). As shown, the interaction between pH and competition, specifically within EMF communities, needs further study. Since substrate colonization and competition for that substrate are integral parts of EM proliferation, understanding how pH affects its success will help predict the outcomes of niche development.

Recently, trait-based ecological approaches have been proven to be useful in defining the environments in which a fungus can survive (Koide et al. 2014), highlighting optimal conditions within those environments (Van Nuland and Peay 2020), and predicting the outcomes of ecological interactions, such as competition (Maherali and Klironomos 2012). Leake and Read (1990) showed that differences in the acid tolerance of extracellular proteinases between two ericoid fungi reflected their respective soil environments. Another fungal trait that greatly affects competitive outcomes is growth rate, as effective substrate colonization is a key mechanism for priority effects in these communities (Fukami et al. 2010). Additionally, EMF communities often appear to be structured by mycelial exploration type (Koide et al. 2014, Moeller et al. 2014, Bui et al. 2020). Luckily, key predictive traits are often phylogenetically conserved (Martiny et al. 2015) and thus vary predictably with phylogeny. As a consequence, phylogenetic similarity can increase competition by intensifying niche overlap, as has been shown in bacterial (Tan et al. 2012), fungal (Taylor et al. 2014), yeast (Peay et al. 2012), and arbuscular mycorrhizal (Maherali and Klironomos 2012) communities. Therefore, phylogenetic relatedness has been suggested as a strong predictor of competitive outcomes. This link between phylogeny and competitive outcomes has not been thoroughly explored in an EMF context, though it is likely that EMF communities are structured similarly.

Therefore, we set out to contrast the efficacy of phylogenetic relatedness versus a trait-based approach to predict EMF responses to competition, pH, and their interaction. We hypothesized that phylogenetic distance would be the best predictor of competition and that all fungi would perform better (have higher growth rates) at a lower pH. In our study, we grew five EMF in single and pairwise culture assays on media at two different pH levels. We found that both phylogenetic relatedness and growth rate predicted the outcomes of competition equally well, which suggests a benefit to considering both approaches in future work.

## Methods

In order to address our hypotheses, we performed an experiment comparing fungal growth on culture plates. We grew fungi in single culture with an intraspecific competitor (self versus self, or SvS, control), and with an interspecific competitor (competition plates) at two different media pH levels (Supplementary Fig. S1). We quantified measures of growth and competitive ability in order to develop a network of competitive outcomes among these EMF.

### Fungal cultures

To compare competitive outcomes across a broad phylogenetic range of EMF, we used five fungal cultures originally isolated from North America and Europe between 1976 and 2019 (Supplementary Table S1): four basidiomycetes (*Amanita muscaria, Hebeloma cylindrosporum, Laccaria bicolor*, and *Paxillus involutus*) and one ascomycete (*Cenococcum geophilum*). All of these fungi are broadly distributed host generalists (LoBuglio 1999, Marmeisse et al. 2004, Geml et al. 2006, Hedh et al. 2008, Plett et al. 2015), and they (or their congeners) likely co-occur on the root systems of trees in nature (Bahram et al. 2011). Prior to our experiment, we maintained cultures on agar plates in a dark cupboard at room temperature (23°C) and transferred them every two months to sustain growth.

### Experimental design

To explore the effects of competition and pH on the growth of EMF, we inoculated these five fungi in a pairwise factorial experiment on two media with differing pH for a total of 20 combinations (10 pairs x 2 pH levels = 20 competition treatments). We placed culture plugs (7/32″ diameter, about 5.6 mm) on plates equidistant from the central axis (Supplementary Fig. S2). Single control plates consisted of a single plug placed in the center of the plate (5 fungi x 2 pH levels = 10 single control treatments). Intraspecific control plates consisted of two plugs of the same fungi placed in the same way as pair plates for a total of 10 combinations (5 fungi x 2 pH levels = 10 SvS control treatments). All treatments were grown with 10 replicates (*n* = 400).

We used Modified Melin-Norkrans (MMN) medium amended with casein hydrolysate and a micronutrient solution (Supplementary Table S2). We autoclaved the phosphate-containing salts and agar/carbon substrates as separate solutions, and mixed afterwards, to prevent the formation of peroxides (Kawasaki and Kamagata 2017). Before autoclaving, we adjusted half the media to pH 5.6 and the other half to pH 7.0 using 1 M HCl and 1 M NaOH. These pH treatments were chosen to reflect soil pH near trees within the University of California’s Sedgwick Reserve, at which ectomycorrhizal communities have been previously shown to respond strongly to soil pH (Runte et al. 2021; Soil Survey Staff).

**Figure 1.**
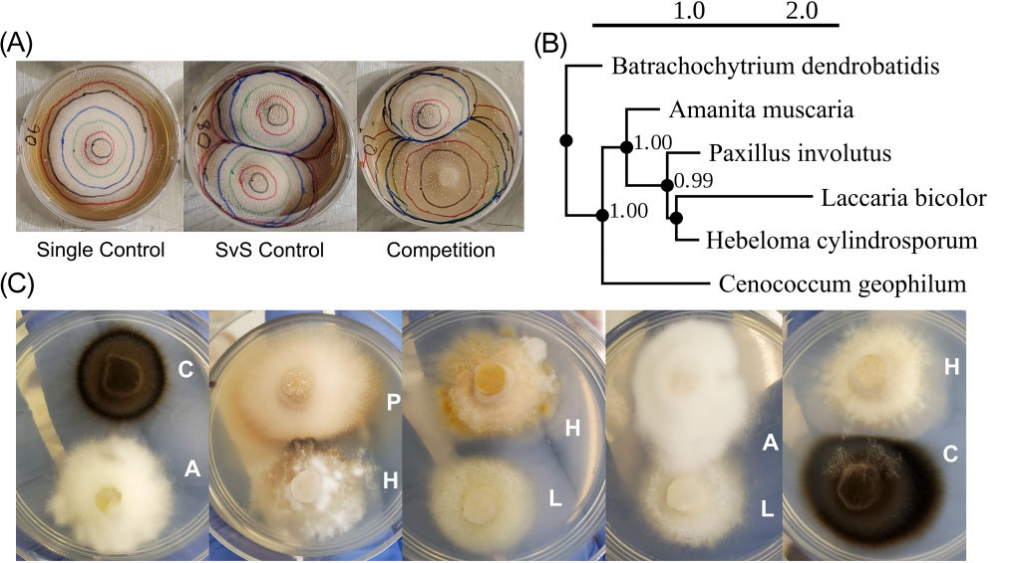
Overview of the experimental design. (A) Examples of the three layouts of experimental plates. Concentric lines denote the colony circumference used to measure growth at different time points. (B) Phylogeny of the five EMF with one outgroup (*Batrachochytrium dendrobatidis*). Numbers on nodes denote posterior probabilities. (C) Examples of variation in growth characteristics of each fungal species [A = *A. muscaria*, C = *C. geophilum*, H = *H. cylindrosporum*, L = *L. bicolor*, P = *P. involutus*].

We stored assay plates in darkness at room temperature and took growth rate measures by regularly tracing the colony circumference (Fig. 1A). Throughout the experiment, we removed plates exhibiting irregular growth, including contamination, a dislodged plug, or dormancy (Supplementary Fig. S3). We took pictures of all plates at the end of the experiment and used the area tool in the software ImageJ (Schneider et al. 2012) to estimate each area measurement.

### Phylogenetic analysis and distance calculations

In order to determine the phylogenetic distances between fungi, we ran a multigene phylogenetic analysis with one outgroup taxon (*B. dendrobatidis*). We extracted genomic DNA from all fungal cultures, amplified the ITS region with primers ITS1F and ITS4 (Gardes and Bruns 1993), and sequenced amplicons with Sanger sequencing at MCLABS (South San Francisco, CA, USA). We then retrieved sequences for the ITS region of our outgroup species and three additional genes (*TOP1, TEF1*, and *RPB2*) from GenBank (Benson et al. 2013), known to be useful DNA barcoding markers (Lücking et al. 2020). We used MAFFT (Katoh et al. 2002) to align the sequences, estimated the phylogeny with the program PhyML (Guindon et al. 2010) using the TN93 substitution model selected with the Smart Model Selection tool (Lefort et al. 2017), and extracted the patristic distance matrix from the tree for later model calculations. The tree closely resembled published phylogenies of EMF (Kohler et al. 2015). We then used the PhyML tool PRESTO (Guindon and Gascuel 2003) to visualize the phylogeny (Fig. 1B).

### Quantifying competition

To calculate growth rates across control and paired treatments, we fit a linear model to the natural log of colony area as a function of experimental day [*lm(log(area measurement)∼day)*]. To estimate maximum growth rates, we restricted our model fit to the time window of exponential growth (the first 3–4 days of each experiment). We used the measured maximum colony area for colony size analyses. All further analyses were conducted with both the growth rate and colony size metrics, but, for coherence, only the results regarding the growth rate metric were included in the main text (see Colony Size Supplement). Since the SvS control plates consisted of two identical plugs, we randomly chose one of the plugs for further analysis.

To quantify the effects of interspecific competition, we calculated the effect of competition (EoC) metric as the ratio of growth rate on competition plates versus SvS control plates [(*growth in competition*/*growth on SvS control plate)*], modeled after the plant-soil feedback metric (Heinze et al. 2016). We then modeled EoC as a function of fungal ID, competitor ID, and pH treatment [*lm*(EoC ∼ fungal ID * competitor ID * pH treatment)] to test for significant interactions between these parameters (Supplementary Table S8).

Since both phylogenetic distance and EoC values are pairwise metrics, we computed a pairwise distance metric for our growth measures. This allowed for more logical models assessing the predictive power of growth metrics on EoC and comparing the difference in efficacy of growth metric versus phylogenetic-based models. Specifically, we calculated the difference between the mean SvS control growth rate in both pH conditions for each pair of fungi (i.e. growth rate distance*_A. muscaria_*_&_*_C. geophilum_* = |mean(*A. muscaria* SvS growth rates in pH 7) − mean(*C. geophilum* SvS growth rates in pH 7)|).

To classify the type of interaction at the front where competing fungi met, we also calculated an index of antagonism (IoA) score based on methods in Wicklow and Hirschfield (1979) (Supplementary Fig. S4). This metric assigns a point value to the intensity of antagonism one fungus exhibits on its opponent from observation of physical distance and overgrowth at the contact zone. For example, a higher point value is awarded to a fungus that grows on top of its opponent after meeting in the middle, while the fungus that is overtaken receives a low score (Supplementary Fig. S4B). We used this as a proxy for the effectiveness of a fungus’s competitive ability.

To visualize the observed competitive hierarchical interactions, we created network plots as complete directed graphs with the R package *visNetwork* (Almende et al. 2019). We calculated the thickness of the control network edges as the growth rate of the fungi over the growth rate of the hypothetical opponent. We calculated the thickness of the EoC network edges as the percentage of times that a fungus had a larger EoC value than its opponent across all replicate plates. Finally, we calculated the thickness of the IoA network plots as the average IoA score of the plugs in each pairing.

### Statistical analysis and modeling

We conducted analysis of variance tests (ANOVA) and Tukey Honest Significant Differences (TukeyHSD) tests to find the differences in growth measures between (1) pH treatments for single controls, (2) pH treatments for SvS controls, (3) pH treatments for competition plates, and (4) control and competition plates under the same pH (Supplementary Table S3–7).

To investigate whether phylogenetic distance or growth rate better explained a strain’s response to competition, we built linear mixed effects models using the *lme4* package (Bates et al. 2015). Each model predicted the EoC on growth rate using pH as a fixed effect along with either phylogenetic distance or growth rate distance, and with fungal identity and plate identity as crossed random effects. For the growth rate distance model, because each pair of fungi had a unique growth rate distance at each pH value, the pH:growth rate distance interaction term was excluded; for phylogenetic distance, the distance value did not vary with pH, so the interaction term was retained: [*lmer(log(EoC) ∼ growth rate distance + pH + (1|fungal ID) + (1|plate ID))*] or [lmer(log(EoC) ∼ phylogenetic distance * pH + (1|fungal ID) + (1|plate ID))]. Due to the limited number of species in our phylogeny, we additionally ran the linear mixed effects models without *C. geophilum* and then again without *C. geophilum* and *P. involutus* to investigate their robustness. We compared these models using a chi-square test computed by the *stargazer* function from the *Stargazer* package (Hlavac 2018). We then used the package *lmerTest* (Kuznetsova et al. 2017) to generate *P*-values for each of the models and the *r.squared.GLMM* function, from the package *MuMIn* (Bartoń 2020), to generate pseudo-R^2^ values for the model equations. To summarize and visualize these data, we created summary tables for our models with the package *Stargazer*, and we used the package *ggeffects* to plot our models (Lüdecke 2018). Additionally, to make sure our linear mixed effects models did not have correlated main effects, we modeled the correlation between growth rate distance and phylogenetic distance [*lmer(phylogenetic distance ∼ growth rate distance + (1|pH)*]. We performed all analyses in R (version 4.1.1) (R Core Team 2021) using the RStudio interface (version 1.4.1717) (RStudio Team 2021).

## Results

### pH significantly affects EMF growth

**Figure 2.**
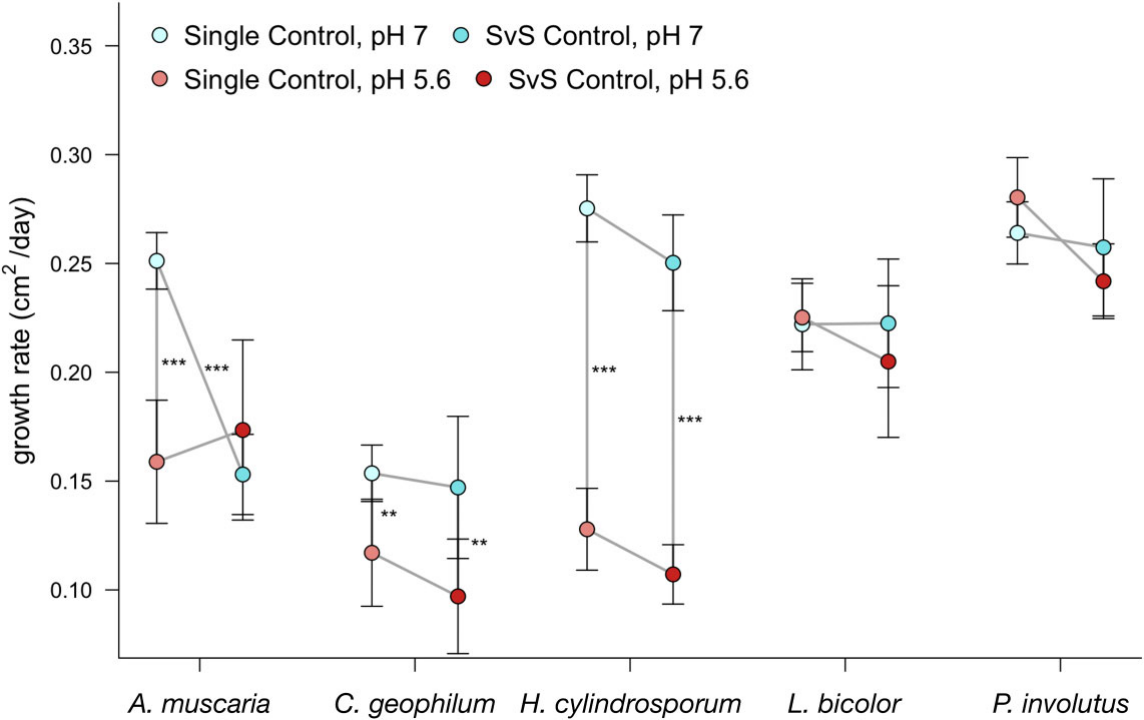
Fungal responses to growth medium pH and intraspecific competition. Growth rate of single and SvS controls for *A. muscaria, C. geophilum, H. cylindrosporum, L. bicolor*, and *P. involutus* at two pH levels. Lines connecting points represent useful comparisons. Error bars represent standard error. Asterisks next to lines represent significant differences in growth rate in that comparison. [All significant differences were determined by Tukey HSD tests; “***” *P* < 0.001, “**” *P* < 0.01, “*” *P* < 0.05].

Regular (e.g. uncontaminated, positive) growth (Supplementary Fig. S3) was observed on 368 out of the total 400 plates (92%). Fungal growth rates were significantly affected by pH in both single (ANOVA: *F_1,89_* = 19.35, *P =* 3.01e^−05^) and SvS (ANOVA: *F_1,94_* = 11.92, *P =* 8.34e^−04^) controls. In the single controls, the growth rates for *A. muscaria, C. geophilum*, and *H. cylindrosporum* were all significantly lower under more acidic conditions (Fig. 2), while the other two fungi (*L. bicolor* and *P. involutus*) were much less affected by pH. The SvS controls showed similar results, except that *A. muscaria* additionally grew poorly in the neutral pH condition (Fig. 2). Although most tested fungi grew poorly on the acidic media relative to the neutral media, *H. cylindrosporum* was the most dramatically affected. Its average growth rate on the acid media was 46.4% of its rate when grown in single culture and 42.8% when grown in intraspecific competition. By contrast, the growth of *P. involutus* was unaffected by media pH in both control treatments (Fig. 2). The growth of the five fungi on the SvS plates was used to model single species growth rates, from which we calculated growth rate distances for later modeling.

### The effects of competition vary with fungal identity and pH

**Figure 3.**
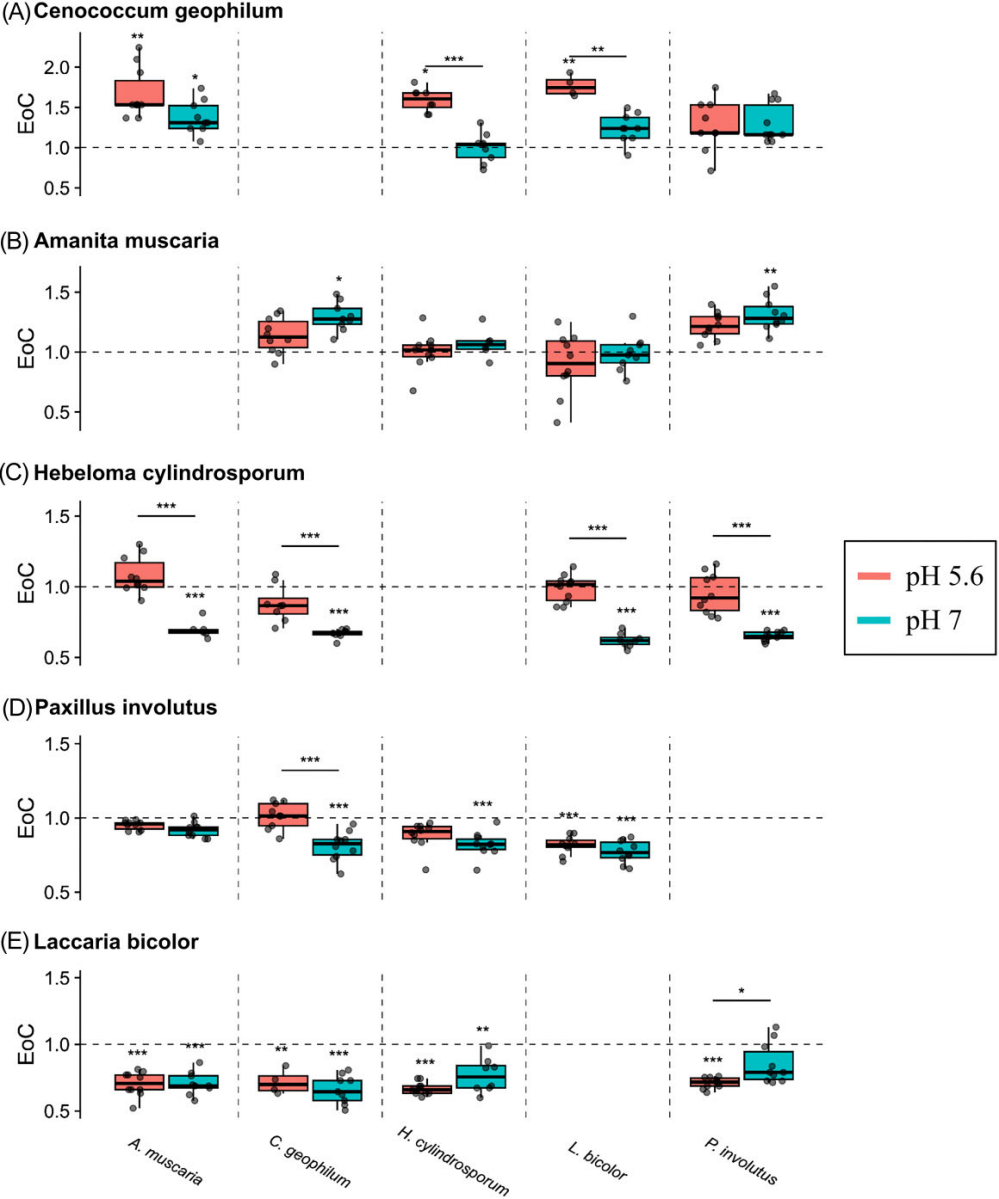
Fungal responses to growth medium pH and interspecific competition. The EoC (effect of competition) metric, calculated as the log ratio of growth rate on competition plates vs. control plates [(growth in competition/growth on control plate)], differs depending on the pH in which the competition occurs [pH 5.6 (red), pH 7 (blue)]. Fungi are ordered from most competitive (top) to least competitive (bottom). Opponent fungi are denoted at the bottom: *A. muscaria* (left), *C. geophilum* (middle left), *H. cylindrosporum* (middle), *L. bicolor* (middle right), and *P. involutus* (right). Asterisks above the box plots represent significant differences in growth rate between the control and competition plates. Asterisks above the bars represent significant differences in growth rate between pH treatments. [All significant differences were determined by Tukey HSD tests; “***” *P* < 0.001, “**” *P* < 0.01, “*” *P* < 0.05].

When grown in interspecific competition, fungal growth rates varied in ways that depended on the identity of the fungus, its competitor, and the pH of the plate (linear model: *F_39,322_* = 34.16, *P =* 2.20e^−16^, adjusted R^2^ = 0.782, Supplementary Table S8). Most fungi grew more slowly when competing with other strains, with *L. bicolor* being the most consistently inhibited by competition (Fig. 3D). This pattern was far from universal, however: the growth rate of *A. muscaria* was mostly unaffected by the presence of competitors (Fig. 3A), while *C. geophilum* generally grew faster in the presence of other species relative to its controls (Fig. 3B). Intriguingly, *H. cylindrosporum* responded differently to competition depending on the pH of the media: at pH 7, it was negatively affected by competition regardless of competitor identity, while at pH 5.6, it grew slightly faster when competing with *A. muscaria* than when growing on its own (Fig. 3C).

### Competitive networks reveal complex interactions and lack of hierarchical structure

**Figure 4.**
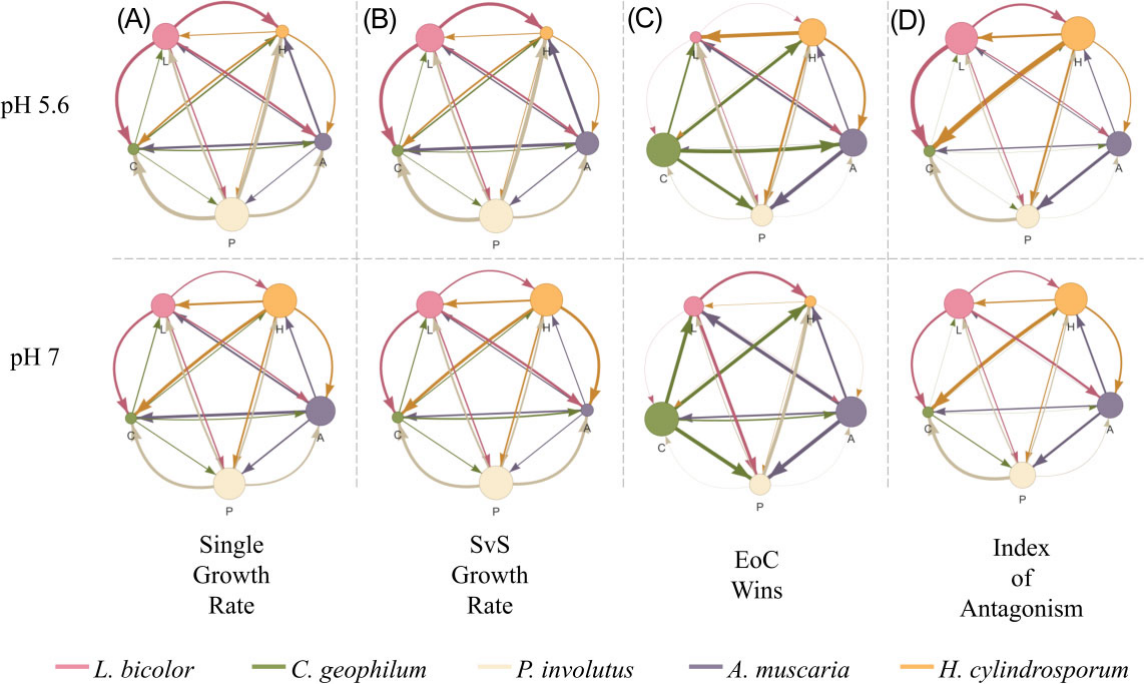
Fungal competitive networks vary by metric and shift with growth medium pH. Network plots depict average interactions between fungi based on four measures: single control growth rate (A), SvS control growth rate (B), EoC wins (C), and the IoA score (D). The width of arrows for each measure was calculated, respectively, as the ratio of the mean growth rate over the mean growth rate of the theoretical opponent (for both controls), the number of competition plates on which a fungus had a higher EoC value than its opponent, and the average IoA score for each fungus in interspecific competition. The size of the nodes is proportional to the sum of all outgoing arrow widths [pink = *L. bicolor*, green = *C. geophilum*, beige = *P. involutus*, purple = *A. muscaria*, orange = *H. cylindrosporum*; pH 5.6 (top) and pH 7 (bottom)].

The network plots highlight the complexity of the competitive interactions between the five fungi. Based on the results of the control plates, *L. bicolor* and *P. involutus* seem to be the highest performers at pH 5.6, and *P. involutus* and *H. cylindrosporum* at pH 7 (Fig. 4A and B). These results are inconsistent with fungal performance under competition. At pH 5.6, *L. bicolor* was most negatively affected by competition, while *C. geophilum* was dominant in terms of positive performance under competitive stress (Fig. 4C). At the neutral pH, *H. cylindrosporum* becomes the weakest performer, and *A. muscaria* joins *C. geophilum* at the upper end of the performance spectrum (Fig. 4C).

In certain pairings, the outcome of competition switched based on what pH environment the competition took place in. Under the more acidic condition, both *P. involutus* and *H. cylindrosporum* outcompeted *L. bicolor*. This outcome reversed for both pairings in the neutral condition (Fig. 4C), with *L. bicolor* instead outperforming the other two fungi. Furthermore, *H. cylindrosporum* outgrew *A. muscaria* at pH 5.6, while at pH 7, *A. muscaria* was the dominant fungus (Fig. 4C). These results are inconsistent with the predictions from the control plates, which suggest that *L. bicolor*, under pH 5.6, should have a faster growth rate than *H. cylindrosporum*, and *H. cylindrosporum*, under pH 7, should have a faster growth rate than *A. muscaria* (Fig. 4A and B).

The IoA metric showed little variance between the pH treatments (Fig. 4D). *Hebeloma cylindrosporum* remained dominant, while *C. geophilum* remained the weakest competitor. The performance of the other three fungi was all similar between pH conditions.

### Both growth rate and phylogenetic models have similar predictive efficacy

**Figure 5.**
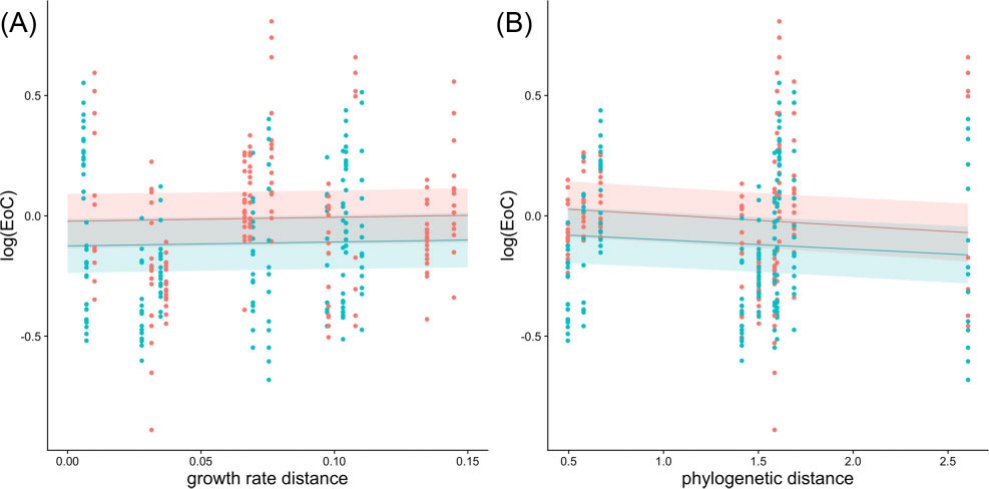
Phylogenetic distance and growth rate distance model predictions on EoC. (A) Graphical results of the linear mixed effects model when using growth rate distance to predict the effects of competition on growth rate. (B) Graphical results of the linear mixed effects model when using phylogenetic distance to predict the effects of competition on growth rate. [pH 5.6 (red), pH 7 (blue); ribbons represent standard error].

We found that both phylogenetic distance and growth rate distance predicted the effects of competition with similar efficacy (ANOVA: AIC_growth rate dist_ = −139.516, AIC_phylogenetic dist_ = −131.227, Chi-sq = 0, *P* = 1, Table 1). In both models, pH had a significant effect on the correlation between predictive and response variables (growth rate distance model: estimated value = −0.103, *P* = 2.05e^−06^; phylogenetic distance model: estimated value = −0.111, *P* = 0.033, Fig. 5). Additionally, we found that growth rate distance and phylogenetic distance were not significantly correlated (linear model: *P* = 0.791) (Supplementary Table S9), allowing us to conclude that these variables can be treated independently. When investigating models with reduced taxa, we similarly found that there were no differences in predictive power between the two models (Supplementary Table S10–11).

**Table 1.** ANOVA table comparing the two linear mixed effects models.

	Dependent variable:	
	EoC	
	Growth rate-based	Phylogenetic-based
	(1)	(2)
Growth rate distance	0.163	
	(0.258)	
Phylogenetic distance		−0.046
		(0.029)
pH 7	−0.102***	−0.111*
	(0.021)	(0.052)
Phylogenetic distance:pH 7		0.007
		(0.036)
Constant	−0.022	0.050
	(0.113)	(0.120)
Conditional pseudo-R2	0.6672	0.6159
Observations	362	362
Log likelihood	75.758	72.613
Akaike inf. crit.	−139.516	−131.227
Bayesian inf. crit.	−116.166	−103.985

*Note:* *P < 0.05; **P < 0.01; ***P < 0.001. The first column depicts summary statistics for the model that uses growth rate distance to predict the EoC on growth rate. The second column depicts summary statistics for the model that uses phylogenetic distance to predict the EoC on growth rate. Numbers in parentheticals denote standard error.

### Colony size growth metric produces similar results

Fungal colony sizes were significantly affected by pH in both single (ANOVA: *F_1,89_* = 38.45, *P =* 1.78e^−08^) and SvS (ANOVA: *F_1,94_* = 30.90, *P =* 2.53e^−07^) controls. When grown in competition, fungal colony sizes varied in ways that depended on the identity of the fungus, its competitor, and the pH of the plate (linear model: *F_39,322_* = 35.04, *P =* 2.20e^−16^, adjusted R^2^ = 0.786, Colony Size Supplementary Table S7). The network plots made using the colony size metric still show a lack of hierarchical structure, though *H. cylindrosporum* replaces *C. geophilum* as the dominant fungus when considering EoC wins (see Colony Size Supplement). Results differ most when considering the linear mixed effects models. For colony size, we found that phylogenetic distance predicted the effects of competition better than colony size distance (ANOVA: AIC_colony size dist_ = 300.651, AIC_phylogenetic dist_ = 290.172, Chi-sq = 12.479, *P* = 4.12e^−04^). When investigating models with reduced taxa, we found that with four taxa (without *C. geophilum*), there were no differences in predictive power between the two models, and with three taxa (without *C. geophilum* and *P. involutus*), phylogenetic distance again predicted the effects of competition better than colony size distance (see Colony Size Supplement).

## Discussion

Outcomes of EM fungal competition can alter forest ecology at both individual (e.g. tree health) and landscape-wide (e.g. biogeochemical cycling) scales. However, few studies of EM competition (compared to the high diversity of fungal taxa co-occurring on tree root systems) exist from which to derive predictions of competitive outcomes. Therefore, we set out to compare two predictive methods of understanding how EM fungi perform in competition. Our results suggest that incorporating either phylogenetic relatedness or a growth measure approach may provide useful insight in predicting the effects of competition on fungal performance. Overall, while pH did strongly influence competitive performance, interactions between the five isolates were complex, highlighting the need for a better understanding of fungal competitive mechanisms and their reaction to environmental changes. In this way, our findings parallel other studies of fungal competition that show context-dependent outcomes (Kennedy 2010, Mujic et al. 2016) that can vary with fungal growth habits and environmental tolerances (Marín et al. 1998, Chen and Chou 2017, Maynard et al. 2019).

While, in our experiment, phylogenetic distance predicted competitive interactions equally well to the growth rate metric, there is much debate in the literature surrounding this topic. Some suggest that trait-based approaches should be most effective in predicting these kinds of outcomes (Mahon et al. 2021) because traits relevant to competition may vary across lineages in ways that are poorly captured by the underlying phylogeny (Cadotte et al. 2017). However, others argue for combining approaches (Mayfield and Levine 2010, Cadotte et al. 2017) because it is difficult to experimentally address the entire scope of ecologically relevant traits and their individual roles in shaping competitive outcomes (Cadotte 2013). To address this conflict, Mayfield and Levine (2010) posit that phylogenetic relatedness would positively correlate to the effects of competition only if phylogenetic structure captured differences in niche preferences such that closely related species more harshly exclude one another. Alternatively, if phylogenetic relatedness was correlated to differences in competitive traits, one would expect closely related species to have similar competitive advantages and therefore outcompete distantly related species (Mayfield and Levine 2010). Our results support the second hypothesis, as phylogenetic distance negatively correlated with the EoC metric, meaning that more distantly related species experienced stronger negative competitive effects. Finally, though our work suggests that both trait and phylogenetic models have similar competence, future research may find that increasing the number of relevant traits studied will provide a substantially more powerful predictor for competitive effects.

The differences in efficacy between our two growth measures, growth rate and colony size, in predicting competitive outcomes further illustrate the complex nature of interspecific interactions. We found that the effects of competition on growth rate were predicted equally well by initial growth rate and phylogenetic distance, but phylogeny was the better predictor of final colony size (Colony Size Supplementary Table S1). In an ecological context, these growth measures can represent a proxy for two types of competition: initial growth rate, representing exploitation competition, and final colony size, representing interference competition (Boddy and Hiscox 2016, Smith et al. 2018). While an organism’s ability to colonize its environment, determined in part by growth rate, may be less dependent on phylogenetic distance, we found that the longer-term outcome of the physical interaction between competing fungi (colony size) was more dependent on phylogeny, perhaps because phylogeny predicts unmeasured traits like chemical defenses or the close proximity of the fungi on Petri dishes intensifying interference competition. This result corroborates previous work that posited the importance of phylogenetic relatedness increases at smaller ecological scales, specifically with individual biotic interactions (Carboni et al. 2013). Both evolutionary relatedness and trait differences may influence competition, but our results suggest their effects depend on the specific competitive outcome measured.

While most competitive outcomes between species pairs were negative, *C. geophilum* experienced significant facilitation in half of its interspecific pairings. As *C. geophilum* is most distantly related to the other four fungi, this finding supports the idea that phylogenetically distant species are more likely to experience facilitative biotic interactions (Valiente-Banuet and Verdú 2007). This finding contradicts the overall trend in our data of intensifying competition with declining relatedness, suggesting that the sign of species interactions may reverse at sufficient phylogenetic distance. Few studies have shown evidence for facilitation in EM fungi (Mamoun and Oliver 1993, Koide et al. 2005, Pickles et al. 2010, Gorzelak et al. 2012), perhaps because mycorrhizal interactions are predominantly studied in the context of competition (Valiente-Banuet and Verdú 2007). Further, the degree of taxonomic variation in studies of species interactions can generally limit the type of interaction that may be detected. Specifically, if the strength of competition scales with phylogenetic relatedness, then studies may need to include species that range in relatedness (including across higher taxonomic ranks) to be able to detect the full spectrum of negative to positive interactions (Philippot et al. 2010). Though our study used five EM fungi with varying degrees of taxonomic relatedness for this reason, we saw few differences between the power of our models to predict competitive interactions when considering taxa of differing relatedness. Future studies constructed in a phylogenetically diverse and nested way can help us to identify the phylogenetic scales at which competitive vs. facilitative interactions dominate. Importantly, our results reflect interactions between only one strain of each species tested. Because intraspecific variation can exceed interspecific variation in some cases (Johnson et al. 2012, Hazard et al. 2017), a definitive test of phylogeny-function relationships would require replication at the strain level. Future work should prioritize characterizing fungal trait variability within species while also examining differences between species.

Although the interactions we observed among these fungal strains provide intriguing insight into belowground competition, extrapolating to field conditions requires an understanding of how competition will change with environmental context. Our data show the complexity of this interplay. We observed significant changes in growth rate in both control treatments (Fig. 2) and EoC values (Fig. 3) based on pH, with a higher control growth rate in the neutral condition. The maintenance of intracellular pH (Bignell 2012) and modification of environmental pH (Krishna Sundari and Adholeya 2003) are important functions in fungi and involve an arsenal of enzymes, transporters, and signaling molecules. Fungal growth rate can be negatively affected by low pH, as shifts to acidic conditions, even as small as 0.3 pH units, repress phospholipid biosynthesis genes and, therefore, membrane biosynthesis (Kane 2016). We also observed certain competitive outcomes flip between the two pH treatments, which displays the wide range of fungal responses to differing pH. Additionally, the heterogeneity of soil pH likely constrains where particular fungi can grow and so determines the identities of potential competitors. Our data further show that the identity of these competitors is a key determinant of the competitive success of fungi (Fig. 3). Fungi have a plethora of mechanisms by which they can directly and indirectly compete for territory (Boddy and Hiscox 2016). These include strategies of aggression (i.e. secreted organic compounds and antagonistic growth) and defense (i.e. the formation of physical barriers and the upregulation of nutrient acquisition and stress regulatory mechanisms) (Boddy and Hiscox 2016). While our study shows evidence of some of these mechanisms (Supplementary Fig. S1), the specific competitive repertoires of these five EMF are relatively unclear, and further study of EMF competition may reveal mutualism-specific tools. Additionally, in the Petri dish context of this experiment, apparent antagonistic overgrowth could have instead represented more benign coexistence, as long as both fungi still had access to non-limiting media resources. For example, all tested basidiomycetes routinely overgrew *C. geophilum* without clearly impairing its growth (Supplementary Fig. S1).The mycelial morphology of ectomycorrhizal fungi is known to vary with substrate chemistry (Dickie et al. 1998), a process which may have impacted the growth of coinoculated fungi in this experiment. It is difficult to extrapolate from these conditions (i.e. no mycorrhizal association, no beneficial or antagonistic soil microbes) to a field soil, but these fungal interactions are certainly complex and environmentally variable.

Finally, our work suggests that different measures of competition can produce different competitive hierarchies (Fig. 4). Both our EoC and IoA metrics displayed largely different network plots (*C. geophilum* dominant under EoC and *H. cylindrosporum* dominant under IoA), corroborating previous work that posits fungi differentially prioritize competitive and colonizing abilities (Smith et al. 2018). The fact that different fungi are competitively superior, depending on the competition metric used, could help explain the observed diversity of coexisting EMF communities on small spatial scales (Anderson et al. 2014). The complexity of competition and coexistence requires further study, but priority effects, competition-colonization tradeoffs, and niche partitioning are all likely to contribute to ECM community assembly across diverse environments (Kennedy 2010).

To conclude, we tested the usefulness of modeling interspecific interactions between EMF using differences in growth measures and phylogenetic distance by culturing five fungi in single, intraspecific, and pairwise interspecific treatments. Additionally, we replicated our experiment at two pH levels to explore the effect of pH on ECM fungal competitive interactions. Our results show that both phylogeny and growth measures can be useful for predicting the results of EMF competition and that pH strongly influences these interactions. Future research should focus on identifying, which fungal traits most strongly affect biotic interactions and how often variation in these traits is captured adequately by phylogenetic analyses. Furthermore, due to our study design's divergence from the natural EMF ecological context, future research within the scope of ECM competitive interactions should examine how in-vitro observations hold up under the context of the mycorrhizal symbiosis, with a focus on the effects of environmental variables and the presence of facilitative interactions. Since its discovery, the mycorrhizal symbiosis has proven to be an integral component of forest community structure. The complexity of the biotic and abiotic interactions in both the development and maintenance of this symbiosis requires much further study, but doing so will allow us to apply a more informed conceptual framework to the structuring of terrestrial ecological communities.

## Supplementary Material

fiad108_Supplemental_Files

## Data Availability

Data and R code for all analyses are available in a public repository on GitHub (https://github.com/ahsmith22/FungalFightClub). Fungal ITS sequence data can be found in the GenBank database supported by the National Center for Biotechnology Information under accession numbers OP348915- OP348919.
