# Supplementary material for: Fungal Fight Club: phylogeny and growth rate predict competitive outcomes among ectomycorrhizal fungi
Source: FEMS Microbiol Ecol. 2023 Sep 11;99(10):fiad108. doi: 10.1093/femsec/fiad108 (PMC10516346; doi:10.1093/femsec/fiad108)
Supplement: fiad108_Supplemental_Files [file fiad108_supplemental_files.zip › Colony_Size_Supp_data.docx]

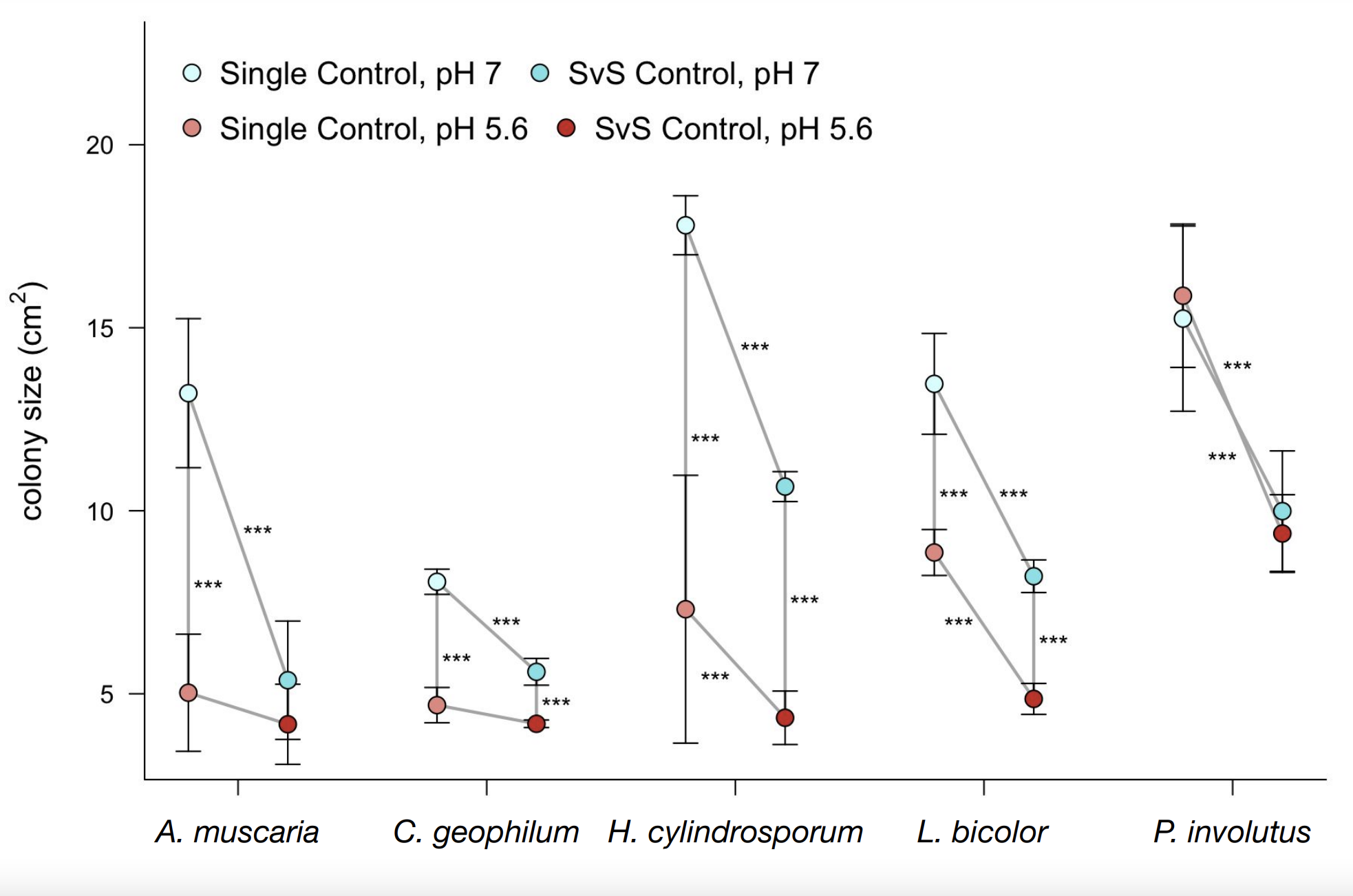


**Colony Size Supplemental Figure 1.** Fungal responses to growth medium pH and intraspecific competition

Colony size of single and SvS controls for *A. muscaria*, *C. geophilum*, *H. cylindrosporum*, *L. bicolor*, and *P. involutus* at two pH levels. Lines connecting points represent useful comparisons. Error bars represent standard error. Asterisks next to lines represent significant differences in colony size in that comparison. [all significant differences were determined by Tukey HSD tests; ‘***’ p<0.001, ‘**’ p<0.01, ‘*’ p<0.05]


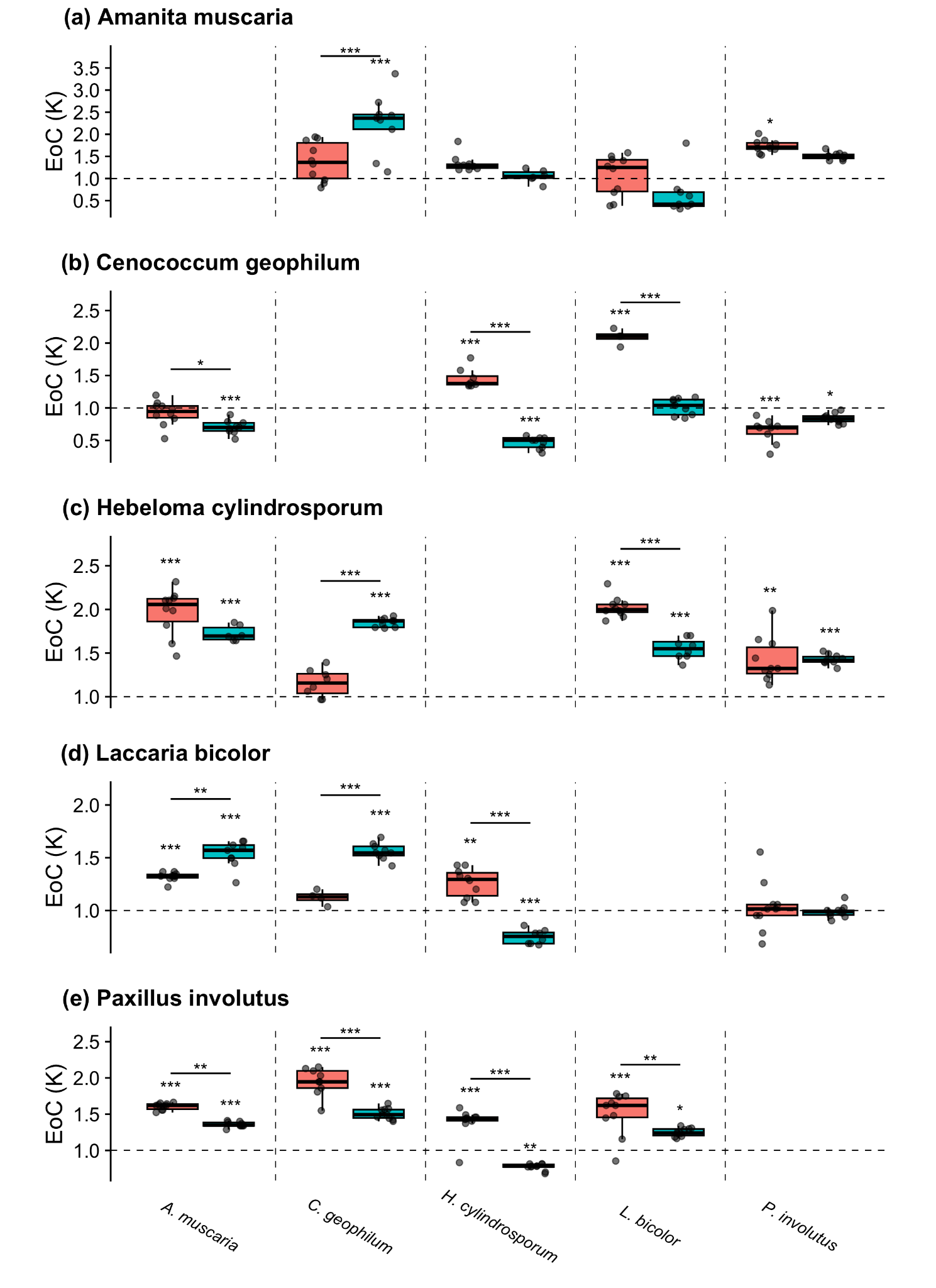


**Colony Size Supplemental Figure 2.** Fungal responses to growth medium pH and interspecific competition

The “effect of competition” metric (EoC), calculated as the log ratio of colony size on competition plates vs control plates [(growth in competition/growth on control plate)], differs depending on the pH in which the competition occurs [pH 5.6 (red), pH 7 (blue)]. Opponent fungi are denoted at bottom: *A. muscaria* (left), *C. geophilum* (middle left), *H. cylindrosporum* (middle), *L. bicolor* (middle right), and *P. involutus* (right). Asterisks above box plots represent significant differences in colony size between the control and competition plates. Asterisks above bars represent significant differences in colony size between pH treatments. [all significant differences were determined by Tukey HSD tests; ‘***’ p<0.001, ‘**’ p<0.01, ‘*’ p<0.05]


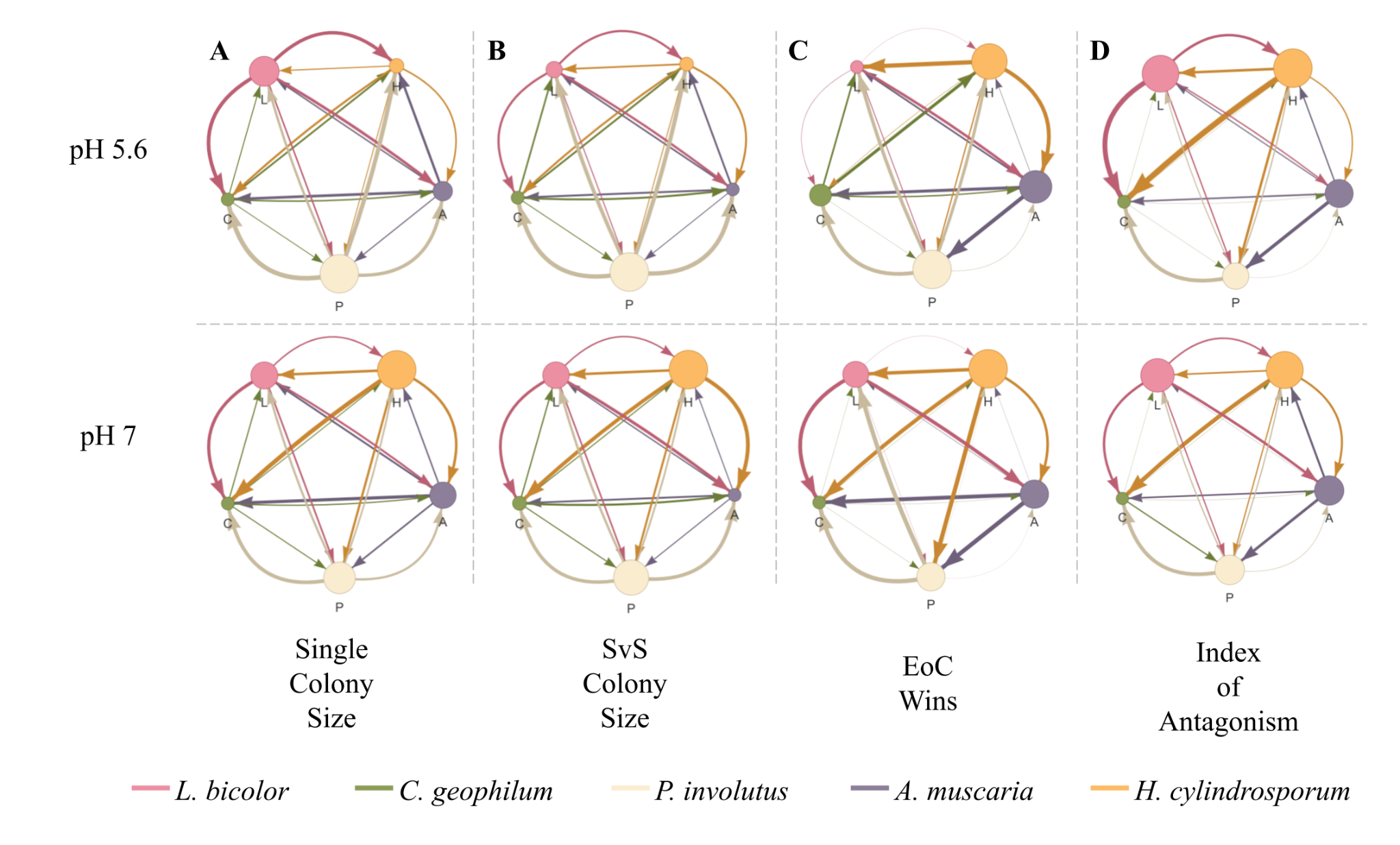


**Colony Size Supplemental Figure 3.** Fungal competitive networks vary by metric and shift with growth medium pH

Network plots depict average interactions between fungi based on four measures: single control colony size **(A)**, SvS control colony size **(B)**, EoC wins **(C)**, and the IoA score **(D)**. The width of arrows for each measure were calculated, respectively, as the ratio of the mean colony size over the mean colony size of the theoretical opponent (for both controls), the number of competition plates that a fungus had a higher EoC value than its opponent, and the average IoA score for each fungi in interspecific competition. The size of the nodes are proportional to the sum of all outgoing arrow widths [pink = *L. bicolor*, green = *C. geophilum*, beige = *P. involutus*, purple = *A. muscaria*, orange = *H. cylindrosporum*; pH 5.6 (top) pH 7 (bottom)].


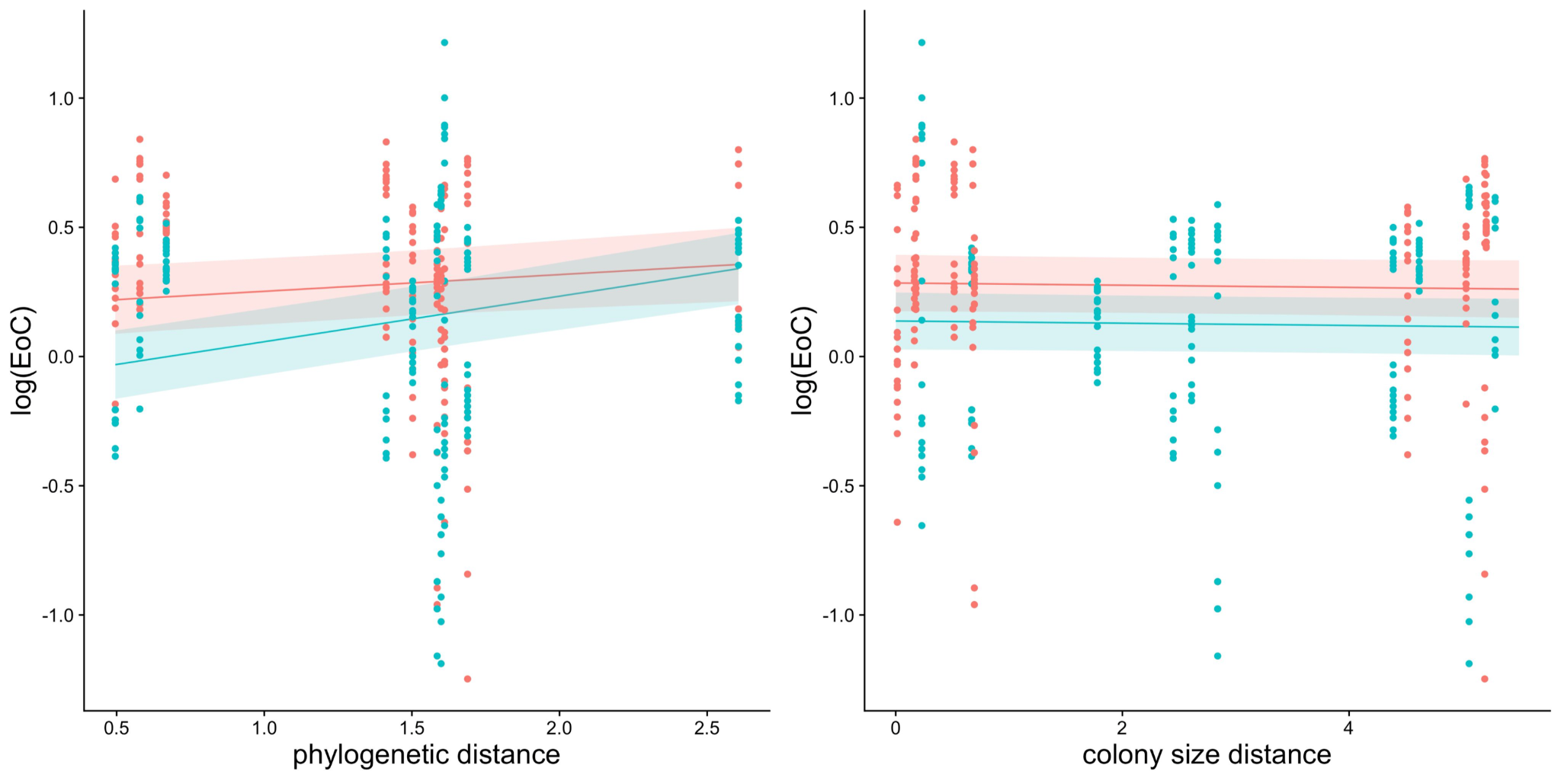


**Colony Size Supplemental Figure 4.** Phylogenetic distance and colony size distance model predictions on EoC

Graphical results of the linear mixed effects model when using phylogenetic distance to predict the effects of competition on colony size **(A)**. Graphical results of the linear mixed effects model when using colony size distance to predict the effects of competition on colony size **(B)**. [pH 5.6 (red), pH 7 (blue); ribbons represent standard error]


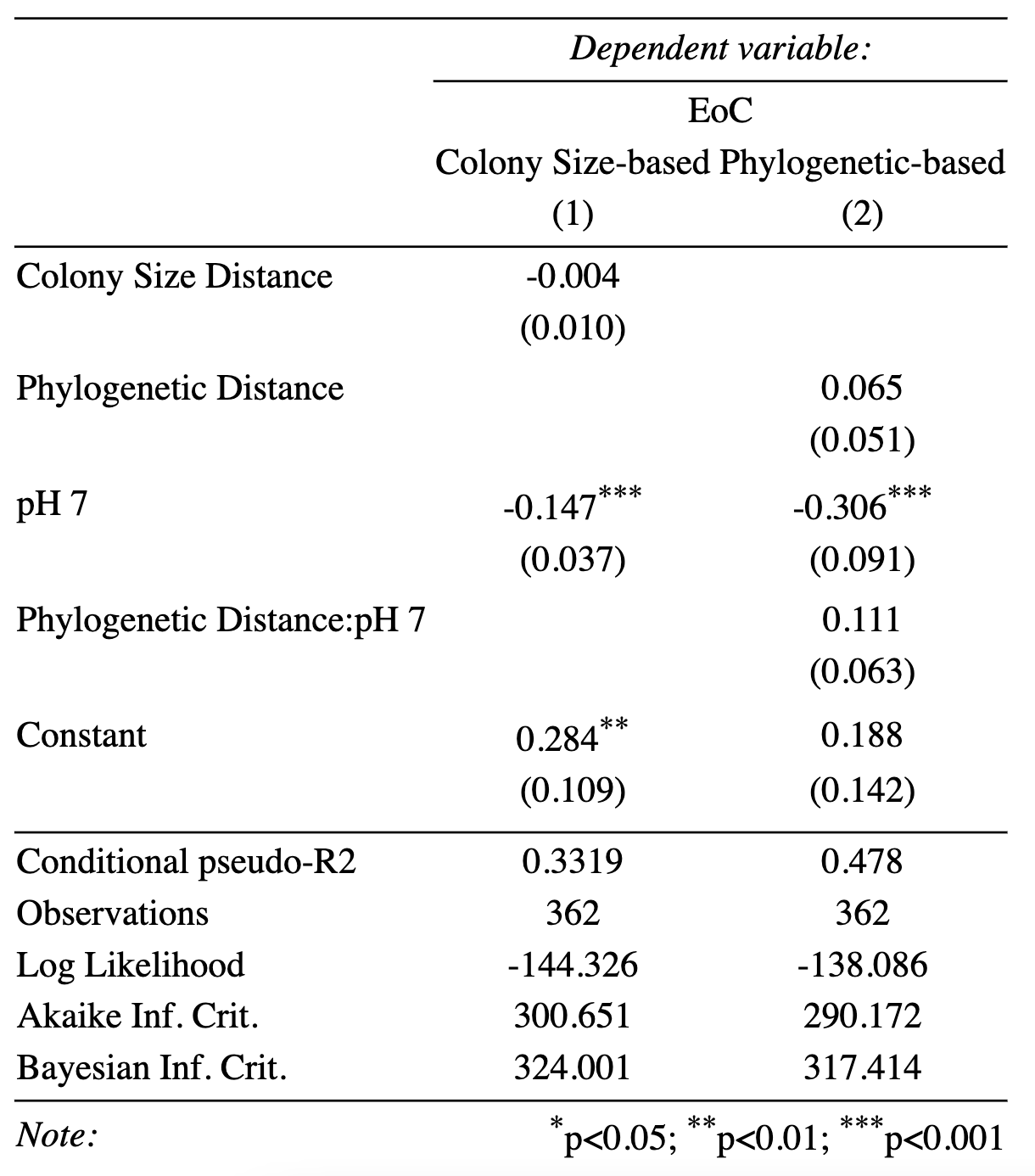


**Colony Size Supplemental Table 1.** ANOVA table comparing the two linear mixed effects models

The first column depicts summary statistics for the model that uses growth rate distance to predict the effect of competition on colony size. The second column depicts summary statistics for the model that uses phylogenetic distance to predict the effect of competition on colony size. Numbers in parentheticals denote standard error.

**Colony Size Supplemental Table 2.** TukeyHSD results comparing effects of pH on single control colony size

| Fungus | Mean Difference | Lower Bound | Upper Bound | Significance |
| --- | --- | --- | --- | --- |
| A | 8.183929 | 5.0734946 | 11.294363 | 5.144385e-11 |
| C | 3.370000 | 0.6822779 | 6.057722 | 3.947746e-03 |
| H | 10.490000 | 7.7286280 | 13.251372 | 2.498135e-11 |
| L | 4.606667 | 1.8452947 | 7.368039 | 2.487948e-05 |
| P | -0.625000 | -3.4757598 | 2.225760 | 9.993568e-01 |

**Colony Size Supplemental Table 3.** TukeyHSD results comparing effects of pH on SvS control colony size

| Fungus | Mean Difference | Lower Bound | Upper Bound | Significance |
| --- | --- | --- | --- | --- |
| A | 1.2033333 | -1.2405088 | 3.647175 | 8.439576e-01 |
| C | 1.4200000 | 0.5819045 | 2.258096 | 1.851302e-05 |
| H | 6.3155556 | 5.0819778 | 7.549133 | 0.000000e+00 |
| L | 3.3511111 | 2.3445421 | 4.357680 | 0.000000e+00 |
| P | 0.6122222 | -1.4689001 | 2.693345 | 9.939549e-01 |

**Colony Size Supplemental Table 4.** TukeyHSD results comparing single and SvS control colony size

| **Fungus** | **pH** | **Mean Difference** | **Lower Bound** | **Upper Bound** | **Significance** |
| --- | --- | --- | --- | --- | --- |
| A | 5 | -0.8619048 | -3.375498 | 1.6516889 | 9.817727e-01 |
| C | 5 | -0.5100000 | -2.740593 | 1.7205931 | 9.991044e-01 |
| H | 5 | -2.9655556 | -5.257272 | -0.6738390 | 2.510602e-03 |
| L | 5 | -4.0000000 | -6.230593 | -1.7694069 | 4.554482e-06 |
| P | 5 | -6.4972222 | -8.920837 | -4.0736076 | 7.714851e-11 |
| A | 7 | -7.8425000 | -9.958925 | -5.7260753 | 2.083653e-10 |
| C | 7 | -2.4600000 | -4.455384 | -0.4646156 | 4.912320e-03 |
| H | 7 | -7.1400000 | -9.190063 | -5.0899374 | 2.083885e-10 |
| L | 7 | -5.2555556 | -7.358875 | -3.1522357 | 3.546899e-10 |
| P | 7 | -5.2600000 | -7.255384 | -3.2646156 | 2.268228e-10 |

**Colony Size Supplemental Table 5.** TukeyHSD results comparing effects of pH on the EoC metric on colony size

| **Fungi** | **Opponent** | **Mean Difference** | **Lower Bound** | **Upper Bound** | **Significance** |
| --- | --- | --- | --- | --- | --- |
| A | C | 0.86717003 | 0.31755221 | 1.41678785 | 1.469948e-04 |
| A | H | -0.28436388 | -0.90208098 | 0.33335322 | 8.347199e-01 |
| A | L | -0.42765136 | -0.97726918 | 0.12196646 | 2.419824e-01 |
| A | P | -0.22663669 | -0.76159540 | 0.30832201 | 8.855769e-01 |
| C | A | -0.21644810 | -0.41522664 | -0.01766956 | 2.355586e-02 |
| C | H | -0.98704687 | -1.19726597 | -0.77682778 | 1.988865e-11 |
| C | L | -1.07462101 | -1.33459767 | -0.81464434 | 1.989264e-11 |
| C | P | 0.19266851 | -0.00611003 | 0.39144704 | 6.390093e-02 |
| H | A | -0.24445906 | -0.51094197 | 0.02202386 | 9.509505e-02 |
| H | C | 0.69271915 | 0.44196783 | 0.94347046 | 8.527745e-11 |
| H | L | -0.47418981 | -0.71897000 | -0.22940963 | 2.163277e-06 |
| H | P | 0.00336818 | -0.22741279 | 0.23414915 | 1.000000e+00 |
| L | A | 0.21174700 | 0.03279458 | 0.39069943 | 9.888353e-03 |
| L | C | 0.43555077 | 0.20150410 | 0.66959743 | 5.644168e-06 |
| L | H | -0.51041461 | -0.69515987 | -0.32566935 | 9.651480e-11 |
| L | P | -0.04384943 | -0.21802892 | 0.13033007 | 9.930839e-01 |
| P | A | -0.25015815 | -0.45953085 | -0.04078544 | 8.636105e-03 |
| P | C | -0.44078012 | -0.65589012 | -0.22567012 | 3.988364e-07 |
| P | H | -0.62010546 | -0.82947816 | -0.41073276 | 0.000000e+00 |
| P | L | -0.26106613 | -0.47043883 | -0.05169343 | 5.154230e-03 |

**Colony Size Supplemental Table 6.** TukeyHSD results comparing effects of interspecific competition on colony size compared to colony size in intraspecific competition

| **Fungi** | **Opponent** | **pH** | **Mean Difference** | **Lower Bound** | **Upper Bound** | **Significance** |
| --- | --- | --- | --- | --- | --- | --- |
| A | C | 5.6 | 1.25333333 | -1.1905088 | 3.69717546 | 8.100914e-01 |
| A | H | 5.6 | 1.07333333 | -1.3705088 | 3.51717546 | 9.148394e-01 |
| A | L | 5.6 | 0.01333333 | -2.4305088 | 2.45717546 | 1.000000e+00 |
| A | P | 5.6 | 2.62333333 | 0.1794912 | 5.06717546 | 2.539018e-02 |
| A | C | 7 | 5.38555556 | 2.9417134 | 7.82939768 | 1.306942e-08 |
| A | H | 7 | -0.33666667 | -3.0833081 | 2.40997476 | 9.999952e-01 |
| A | L | 7 | -2.31444444 | -4.7582866 | 0.12939768 | 7.873780e-02 |
| A | P | 7 | 1.83000000 | -0.5486613 | 4.20866125 | 2.831919e-01 |
| C | A | 5.6 | 0.33000000 | -0.5080955 | 1.16809553 | 9.547031e-01 |
| C | A | 7 | 1.66666667 | 0.8056054 | 2.52772797 | 7.054889e-07 |
| C | H | 5.6 | 1.88250000 | 0.9935654 | 2.77143455 | 5.470392e-08 |
| C | L | 5.6 | 4.57000000 | 3.4613038 | 5.67869618 | 0.000000e+00 |
| C | P | 5.6 | -1.48000000 | -2.3410613 | -0.61893869 | 1.339276e-05 |
| C | H | 7 | -3.01111111 | -3.8721724 | -2.15004980 | 0.000000e+00 |
| C | L | 7 | 0.08888889 | -0.7721724 | 0.94995020 | 9.999989e-01 |
| C | P | 7 | -0.92000000 | -1.7580955 | -0.08190447 | 2.016445e-02 |
| H | A | 5.6 | -3.98555556 | -5.2191333 | -2.75197782 | 0.000000e+00 |
| H | A | 7 | -7.70666667 | -9.0930883 | -6.32024498 | 0.000000e+00 |
| H | C | 5.6 | -0.55555556 | -1.8601310 | 0.74901985 | 9.280428e-01 |
| H | C | 7 | -9.02888889 | -10.2624666 | -7.79531116 | 0.000000e+00 |
| H | L | 5.6 | 4.21555556 | 2.9819778 | 5.44913329 | 0.000000e+00 |
| H | P | 5.6 | 1.67555556 | 0.4419778 | 2.90913329 | 1.210291e-03 |
| H | L | 7 | 5.84000000 | 4.5664904 | 7.11350963 | 0.000000e+00 |
| H | P | 7 | 4.53000000 | 3.3293236 | 5.73067640 | 0.000000e+00 |
| L | A | 5.6 | -1.52000000 | -2.4997223 | -0.54027771 | 1.157233e-04 |
| L | A | 7 | -4.28888889 | -5.3216069 | -3.25617092 | 0.000000e+00 |
| L | C | 5.6 | -0.56500000 | -1.8610508 | 0.73105076 | 9.175171e-01 |
| L | C | 7 | -4.50000000 | -5.5327180 | -3.46728203 | 0.000000e+00 |
| L | H | 5.6 | -1.23000000 | -2.2097223 | -0.25027771 | 3.905249e-03 |
| L | H | 7 | 2.08611111 | 1.0216098 | 3.15061242 | 4.899812e-07 |
| L | P | 5.6 | 0.13000000 | -0.8497223 | 1.10972229 | 9.999903e-01 |
| L | P | 7 | -0.10111111 | -1.1076801 | 0.90545785 | 9.999991e-01 |
| P | A | 5.6 | -5.50222222 | -7.5833446 | -3.42109988 | 3.695744e-10 |
| P | A | 7 | -3.19000000 | -5.2156157 | -1.16438426 | 7.778534e-05 |
| P | C | 5.6 | -8.72222222 | -10.8574087 | -6.58703575 | 3.561764e-10 |
| P | C | 7 | -4.63000000 | -6.6556157 | -2.60438426 | 3.470248e-09 |
| P | H | 5.6 | -3.50222222 | -5.5833446 | -1.42109988 | 1.851768e-05 |
| P | H | 7 | 2.50000000 | 0.4743843 | 4.52561574 | 4.801995e-03 |
| P | L | 5.6 | -4.60222222 | -6.6833446 | -2.52109988 | 9.848724e-09 |
| P | L | 7 | -2.14000000 | -4.1656157 | -0.11438426 | 2.976536e-02 |

**Colony Size Supplemental Table 7.** Linear model results showing the significance of fungal identity, competitor identity, and pH on performance in competition. An “s” in front of the genus represents the focal fungus, while “c” represents the competing fungus. Intercept uses *A. muscaria* and pH 5.6 as a baseline. Test was run under an assumption of alpha = 0.05.

|  | Estimate | Std. Error | t value | Pr(>\|t\|) |
| --- | --- | --- | --- | --- |
| **Intercept** | 2.021 | 0.118 | 17.079 | 5.16E-47*** |
| **s.Cenococcum** | -1.100 | 0.137 | -8.048 | 1.64E-14*** |
| **s.Hebeloma** | -0.052 | 0.137 | -0.378 | 7.06E-01 |
| **s.Laccaria** | -0.699 | 0.097 | -7.237 | 3.39E-12*** |
| **s.Paxillus** | -0.414 | 0.137 | -3.028 | 2.66E-03*** |
| **pH 7** | 0.029 | 0.169 | 0.171 | 8.64E-01 |
| **c.Cenococcum** | -0.638 | 0.137 | -4.667 | 4.50E-06*** |
| **c.Hebeloma** | -0.684 | 0.137 | -5.003 | 9.30E-07*** |
| **c.Laccaria** | -0.954 | 0.137 | -6.983 | 1.66E-11*** |
| **c.Paxillus** | -0.288 | 0.097 | -2.981 | 3.10E-03*** |
| **s.Cenococcum : pH 7** | -0.245 | 0.196 | -1.252 | 2.11E-01 |
| **s.Hebeloma : pH 7** | -0.273 | 0.202 | -1.351 | 1.78E-01 |
| **s.Laccaria : pH 7** | 0.183 | 0.137 | 1.337 | 1.82E-01 |
| **s.Paxillus : pH 7** | -0.279 | 0.195 | -1.434 | 1.52E-01 |
| **s.Hebeloma : c.Cenococcum** | -0.175 | 0.171 | -1.026 | 3.06E-01 |
| **s.Laccaria : c.Cenococcum** | 0.440 | 0.160 | 2.745 | 6.39E-03*** |
| **Fun_IDP : c.Cenococcum** | 0.977 | 0.169 | 5.782 | 1.75E-08*** |
| **s.Cenococcum : c.Hebeloma** | 1.213 | 0.171 | 7.102 | 7.90E-12*** |
| **s.Laccaria : c.Hebeloma** | 0.624 | 0.137 | 4.563 | 7.17E-06*** |
| **s.Paxillus : c.Hebeloma** | 0.468 | 0.167 | 2.794 | 5.52E-03*** |
| **s.Cenococcum : c.Laccaria** | 2.127 | 0.187 | 11.367 | 2.12E-25*** |
| **s.Hebeloma : c.Laccaria** | 1.009 | 0.167 | 6.026 | 4.58E-09*** |
| **s.Paxillus : c.Laccaria** | 0.857 | 0.167 | 5.121 | 5.25E-07*** |
| **s.Cenococcum : c.Paxillus** | 0.013 | 0.139 | 0.092 | 9.27E-01 |
| **s.Hebeloma : c.Paxillus** | -0.258 | 0.137 | -1.889 | 5.98E-02* |
| **pH 7 : c.Cenococcum** | 0.838 | 0.196 | 4.278 | 2.49E-05*** |
| **pH 7 : c.Hebeloma** | -0.313 | 0.202 | -1.548 | 1.23E-01 |
| **pH 7 : c.Laccaria** | -0.457 | 0.196 | -2.330 | 2.04E-02** |
| **pH 7 : c.Paxillus** | -0.256 | 0.139 | -1.845 | 6.60E-02* |
| **s.Hebeloma : pH 7 : c.Cenococcum** | 0.099 | 0.249 | 0.398 | 6.91E-01 |
| **s.Laccaria : pH 7 : c.Cenococcum** | -0.614 | 0.213 | -2.884 | 4.20E-03*** |
| **s.Paxillus : pH 7 : c.Cenococcum** | -1.029 | 0.240 | -4.287 | 2.39E-05*** |
| **s.Cenococcum : pH 7 : c.Hebeloma** | -0.457 | 0.249 | -1.838 | 6.69E-02* |
| **s.Laccaria : pH 7 : c.Hebeloma** | -0.409 | 0.204 | -2.004 | 4.59E-02** |
| **s.Paxillus : pH 7 : c.Hebeloma** | -0.057 | 0.244 | -0.232 | 8.17E-01 |
| **s.Cenococcum : pH 7 : c.Laccaria** | -0.402 | 0.255 | -1.574 | 1.17E-01 |
| **s.Hebeloma : pH 7 : c.Laccaria** | 0.227 | 0.248 | 0.916 | 3.60E-01 |
| **s.Paxillus : pH 7 : c.Laccaria** | 0.446 | 0.239 | 1.866 | 6.30E-02* |
| **s.Cenococcum : pH 7 : c.Paxillus** | 0.665 | 0.197 | 3.370 | 8.44E-04*** |
| **s.Hebeloma : pH 7 : c.Paxillus** | 0.503 | 0.202 | 2.487 | 1.34E-02** |
| Adjusted R2  Residual Std. Error  F Statistic | 0.786  0.216 (df = 322)  35.04*** (df = 39; 322) | | | |
| Note: *p<0.1; **p<0.05; ***p<0.01 | | | | |

**Colony Size Supplemental Table 8.** Anova results showing the comparison between phylogenetic distance and colony size distance models run with four taxa (without *C. geophilum*).

|  | **npar** | **AIC** | **BIC** | **logLik** | **deviance** | **Chisq** | **Df** | **Pr(>Chisq)** |
| --- | --- | --- | --- | --- | --- | --- | --- | --- |
| Colony Size Distance | 6 | 128.87 | 149.39 | -58.435 | 116.87 |  |  |  |
| Phylogenetic Distance | 7 | 132.29 | 156.24 | -59.146 | 118.29 | 0 | 1 | 1 |

**Colony Size Supplemental Table 9.** Anova results showing the comparison between phylogenetic distance and colony size distance models run with three taxa (without *C. geophilum* & *P. involutus*).

|  | **npar** | **AIC** | **BIC** | **logLik** | **deviance** | **Chisq** | **Df** | **Pr(>Chisq)** |
| --- | --- | --- | --- | --- | --- | --- | --- | --- |
| Colony Size Distance | 6 | 84.358 | 100.34 | -36.179 | 72.358 |  |  |  |
| Phylogenetic Distance | 7 | 82.359 | 101.00 | -34.180 | 68.359 | 3.9987 | 1 | 4.55e-02* |
