# Supplementary material for: Fungal Fight Club: phylogeny and growth rate predict competitive outcomes among ectomycorrhizal fungi
Source: FEMS Microbiol Ecol. 2023 Sep 11;99(10):fiad108. doi: 10.1093/femsec/fiad108 (PMC10516346; doi:10.1093/femsec/fiad108)
Supplement: fiad108_Supplemental_Files [file fiad108_supplemental_files.zip › Supp_data Supplemental_Material.docx]

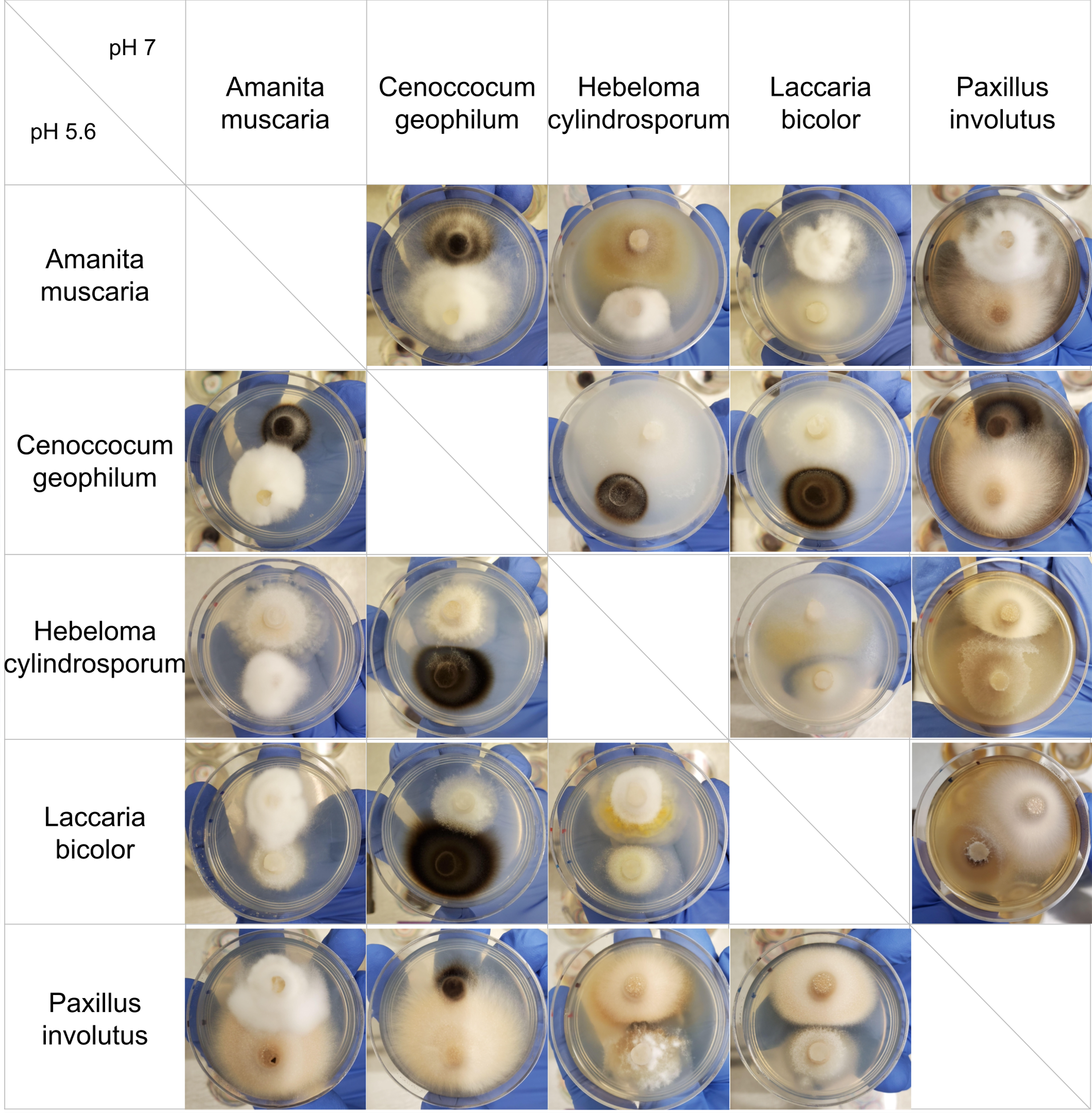


**Supplemental Figure 1.** Example pictures of all experimental conditions. Upper triangle is at pH 7 and lower triangle is at pH 5.6.

##
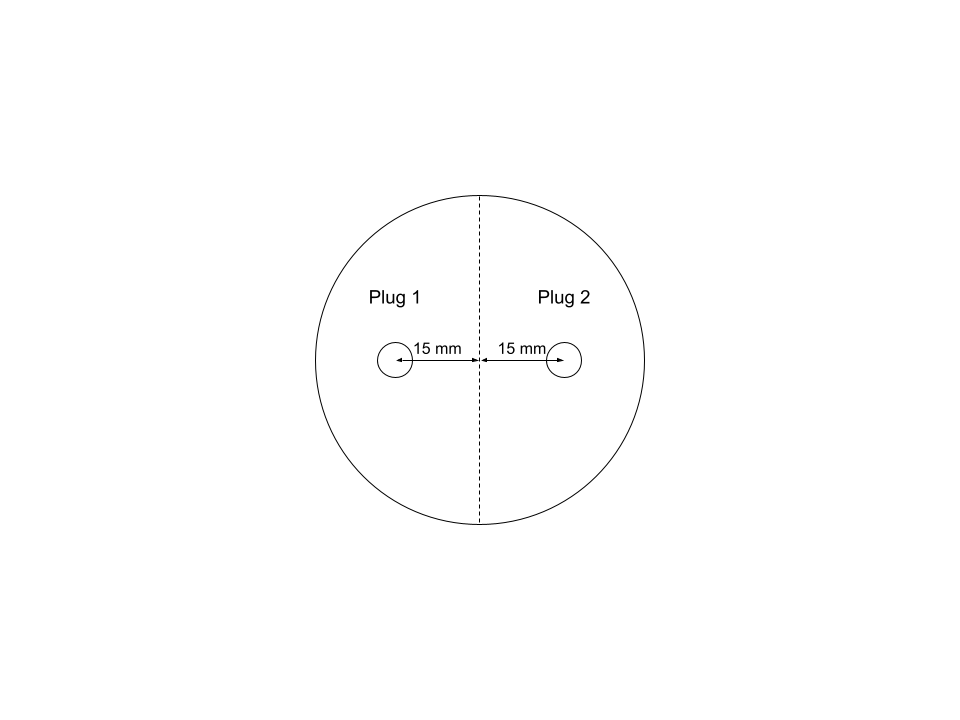


**Supplemental Figure 2.** Diagram of plug layout on plates.


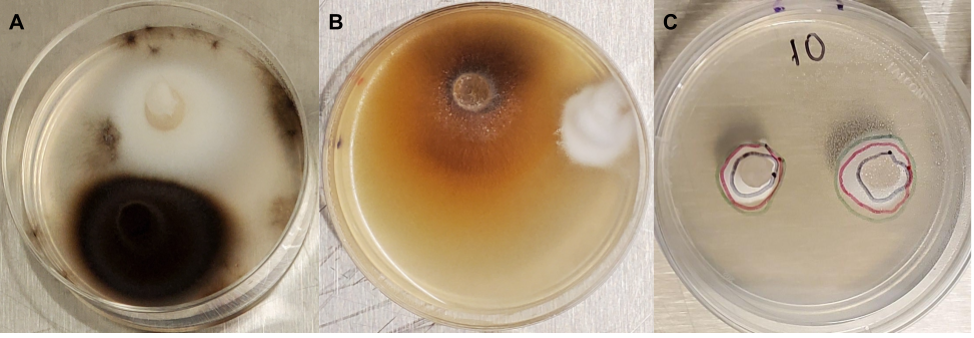


**Supplemental Figure 3.** Examples of irregular plates that were excluded from analyses A) contamination, B) dislodged plug, C) dormancy.


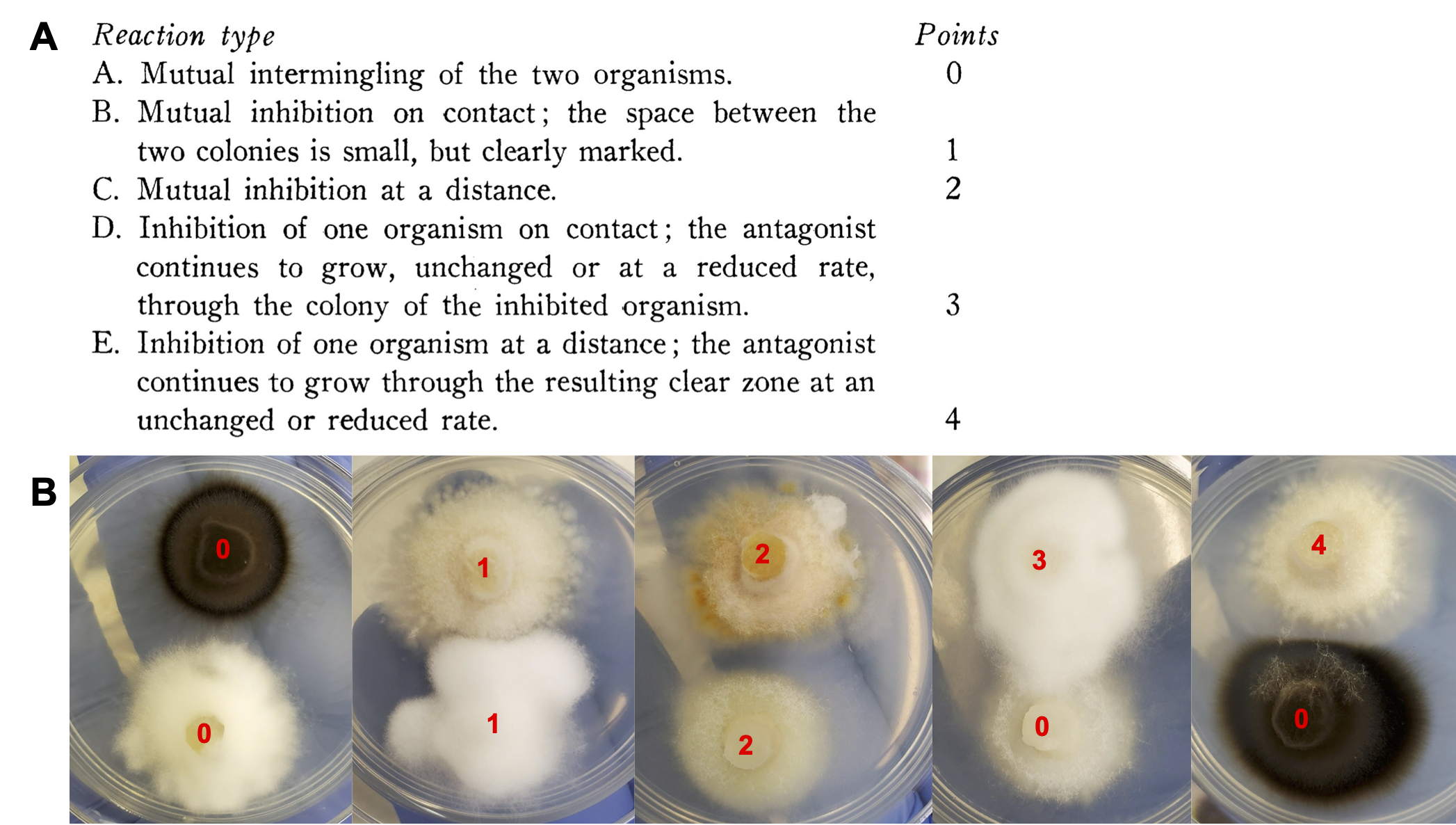


**Supplemental Figure 4.** Reaction type scoring

**A** Scoring rubric from Wicklow & Hirschfield (1979). Each plug on each replicate plate was given a score. Inhibition of growth was determined by visual comparison of the shape of the growing edge of the fungal control to that of the fungi in competition. **B** Example point scores for each reaction type.

| **Supplemental Table 1. Metadata on fungal species used in experiment** | | | | | |
| --- | --- | --- | --- | --- | --- |
| **Fungus name** | **Order** | **Source** | **Strain code from source** | **Strain origin** | **Year of isolation** |
| *Amanita muscaria* | Basidiomycetes | Denny Wang/Anne Pringle | Bigelow - 2 | Bigelow State Park, Connecticut, USA | 2019 |
| *Cenococcum geophilum* | Ascomycetes | Peter Kennedy | Ccg001: AMC fC244 | Cedar Creek Ecosystem Science Reserve, Minnesota, USA | 2015 |
| *Hebeloma cylindrosporum* | Basidiomycetes | Peter Pellitier | TV98 IV3 | France (Atlantic coast) | 1995 |
| *Laccaria bicolor* | Basidiomycetes | Michael Van Nuland | S238N | Crater Lake National Park, Oregon, USA | 1976 |
| *Paxillus involutus* | Basidiomycetes | Michael Van Nuland | ATCC 200175 | Penicuik, Scotland, UK | 1995 |

| **Supplemental Table 2.** Modified Melin-Norkrans agar medium with supplements | |
| --- | --- |
| **Compound** | **Per liter** |
| *Salt solution: (with 460 mL MiliQ water)* |  |
| CaCl₂ | 50 mg |
| NaCl | 25 mg |
| MgSO₄ x 7H₂O | 155 mg |
| (NH₄)₂HPO₄ | 125 mg |
| KH₂PO₄ | 500 mg |
| FeCl₃ x 6H₂O | 7.2 mg |
| thiamine-HCl | 1 mg |
| casein hydrolysate | 1 g |
| *Carbon solution: (with 500 mL MiliQ water)* |  |
| D-glucose | 10 g |
| malt extract powder | 5 g |
| agar | 15 g |
| *Micronutrient solution: (10mL of stock solution)* |  |
| KCl | 37.28 mg |
| H₃BO₃ | 15.46 mg |
| MnCl₂ x 4H₂O | 9.89 mg |
| ZnSO₄ x 7H₂O | 5.75 mg |
| CuSO₄ x 5H₂O | 1.25 mg |
| Na₂MoO₄ x 2H₂O | 0.25 mg |

**Supplemental Table 3.** TukeyHSD results comparing effects of pH on single control growth rate

| Fungus | Mean Difference | Lower Bound | Upper Bound | Significance |
| --- | --- | --- | --- | --- |
| A | 0.092327462 | 0.06107060 | 0.12358433 | 2.528189e-11 |
| C | 0.036529319 | 0.00952030 | 0.06353834 | 1.288009e-03 |
| H | 0.147451917 | 0.11970279 | 0.17520105 | 2.497214e-11 |
| L | -0.003137087 | -0.03088622 | 0.02461204 | 9.999976e-01 |
| P | -0.016322585 | -0.04496998 | 0.01232481 | 6.992224e-01 |

**Supplemental Table 4.** TukeyHSD results comparing effects of pH on SvS control growth rate

| Fungus | Mean Difference | Lower Bound | Upper Bound | Significance |
| --- | --- | --- | --- | --- |
| A | -0.02041536 | -0.06018461 | 0.01935389 | 0.809235775 |
| C | 0.05004318 | 0.01046368 | 0.08962268 | 0.003552973 |
| H | 0.14316358 | 0.12258986 | 0.16373730 | 0.000000000 |
| L | 0.01758757 | -0.01869964 | 0.05387479 | 0.854600488 |
| P | 0.01553957 | -0.01472166 | 0.04580080 | 0.810533243 |

**Supplemental Table 5.** TukeyHSD results comparing single and SvS control growth rates

| Fungus | Mean Difference | Lower Bound | Upper Bound | Significance |
| --- | --- | --- | --- | --- |
| A | 0.0146033659 | -0.02694477 | 0.056151503 | 9.784686e-01 |
| C | -0.0200248545 | -0.05689517 | 0.016845459 | 7.537414e-01 |
| H | -0.0207259251 | -0.05860657 | 0.017154721 | 7.458345e-01 |
| L | -0.0202629716 | -0.05713329 | 0.016607342 | 7.409967e-01 |
| P | -0.0385168616 | -0.07857770 | 0.001543978 | 6.977682e-02 |
| A | -0.0981394557 | -0.13273595 | -0.063542964 | 2.093290e-10 |
| C | -0.0065109951 | -0.03912888 | 0.026106890 | 9.997047e-01 |
| H | -0.0250142641 | -0.05852596 | 0.008497427 | 3.244879e-01 |
| L | 0.0004616896 | -0.03392058 | 0.034843959 | 1.000000e+00 |
| P | -0.0066547062 | -0.03927259 | 0.025963179 | 9.996469e-01 |

**Supplemental Table 6.** TukeyHSD results comparing effects of pH on the EoC metric on growth rate

| Fungus | Opponent | Mean Difference | Lower Bound | Upper Bound | Significance |
| --- | --- | --- | --- | --- | --- |
| A | C | 0.156149429 | -0.071019476 | 0.38331833 | 3.939800e-01 |
| A | H | 0.065142969 | -0.190172840 | 0.32045878 | 9.926332e-01 |
| A | L | 0.094168206 | -0.133000700 | 0.32133711 | 8.965770e-01 |
| A | P | 0.092015881 | -0.129094095 | 0.31312586 | 8.946668e-01 |
| C | A | -0.288518758 | -0.623131027 | 0.04609351 | 1.403472e-01 |
| C | H | -0.594258110 | -0.948128747 | -0.24038747 | 5.074664e-05 |
| C | L | -0.526497764 | -0.964127416 | -0.08886811 | 8.241141e-03 |
| C | P | 0.030412474 | -0.304199795 | 0.36502474 | 9.999914e-01 |
| H | A | -0.382632540 | -0.539397431 | -0.22586765 | 4.033698e-09 |
| H | C | -0.210207735 | -0.357718139 | -0.06269733 | 8.380143e-04 |
| H | L | -0.366498849 | -0.510496597 | -0.22250110 | 1.092681e-09 |
| H | P | -0.298696765 | -0.434459143 | -0.16293439 | 8.300586e-08 |
| L | A | 0.002751392 | -0.140684647 | 0.14618743 | 1.000000e+00 |
| L | C | -0.057002647 | -0.244598470 | 0.13059317 | 9.791722e-01 |
| L | H | 0.100681465 | -0.047397714 | 0.24876064 | 4.062155e-01 |
| L | P | 0.142381830 | 0.002771442 | 0.28199222 | 4.247376e-02 |
| P | A | -0.033152185 | -0.138618010 | 0.07231364 | 9.756239e-01 |
| P | C | -0.204557353 | -0.312913189 | -0.09620152 | 3.128890e-06 |
| P | H | -0.061777663 | -0.167243489 | 0.04368816 | 6.026442e-01 |
| P | L | -0.045200812 | -0.150666637 | 0.06026501 | 8.808428e-01 |

**Supplemental Table 7.** TukeyHSD results comparing effects of interspecific competition on growth rate compared to growth rate in intraspecific competition

| Fungus | Opponent | pH | Mean Difference | Lower Bound | Upper Bound | Significance |
| --- | --- | --- | --- | --- | --- | --- |
| A | C | 5.6 | 0.013280486 | -0.0264887629 | 0.0530497350 | 9.848421e-01 |
| A | H | 5.6 | -0.008603949 | -0.0483731979 | 0.0311653000 | 9.994270e-01 |
| A | L | 5.6 | -0.026764081 | -0.0665333300 | 0.0130051680 | 4.730690e-01 |
| A | P | 5.6 | 0.026730909 | -0.0130383399 | 0.0665001580 | 4.748851e-01 |
| A | C | 7 | 0.042457589 | 0.0026883404 | 0.0822268383 | 2.684918e-02 |
| A | H | 7 | 0.008571367 | -0.0361254100 | 0.0532681443 | 9.997858e-01 |
| A | L | 7 | -0.003767441 | -0.0435366900 | 0.0360018079 | 9.999995e-01 |
| A | P | 7 | 0.045160657 | 0.0064521128 | 0.0838692018 | 9.996464e-03 |
| C | A | 5.6 | -0.052393854 | -0.0919733546 | -0.0128143532 | 1.823145e-03 |
| C | A | 7 | -0.045864866 | -0.0865289376 | -0.0052007948 | 1.496942e-02 |
| C | H | 5.6 | 0.045643918 | 0.0036635180 | 0.0876243180 | 2.231713e-02 |
| C | L | 5.6 | 0.061343644 | 0.0089848865 | 0.1137024024 | 9.592377e-03 |
| C | P | 5.6 | 0.016527278 | -0.0241367935 | 0.0571913493 | 9.450494e-01 |
| C | H | 7 | -0.007470926 | -0.0481349977 | 0.0331931450 | 9.998462e-01 |
| C | L | 7 | 0.026528351 | -0.0141357204 | 0.0671924224 | 5.162468e-01 |
| C | P | 7 | 0.034503252 | -0.0050762492 | 0.0740827522 | 1.416671e-01 |
| H | A | 5.6 | -0.006957888 | -0.0275316102 | 0.0136158346 | 9.832958e-01 |
| H | A | 7 | 0.076809290 | 0.0536864224 | 0.0999321579 | 0.000000e+00 |
| H | C | 5.6 | 0.014558444 | -0.0071993844 | 0.0363162719 | 4.802949e-01 |
| H | C | 7 | 0.083983354 | 0.0634096314 | 0.1045570762 | 0.000000e+00 |
| H | L | 5.6 | -0.002865616 | -0.0234393384 | 0.0177081064 | 9.999854e-01 |
| H | P | 5.6 | -0.006787538 | -0.0273612600 | 0.0137861848 | 9.859155e-01 |
| H | L | 7 | -0.095924255 | -0.1171639648 | -0.0746845442 | 0.000000e+00 |
| H | P | 7 | -0.088306959 | -0.1083319499 | -0.0682819681 | 0.000000e+00 |
| L | A | 5.6 | 0.064254228 | 0.0289348441 | 0.0995736119 | 3.403092e-06 |
| L | A | 7 | 0.066436245 | 0.0292063456 | 0.1036661446 | 5.452057e-06 |
| L | C | 5.6 | 0.061480085 | 0.0147569319 | 0.1082032381 | 1.985863e-03 |
| L | C | 7 | 0.076566015 | 0.0393361155 | 0.1137959145 | 1.254650e-07 |
| L | H | 5.6 | 0.071392050 | 0.0360726659 | 0.1067114336 | 2.064129e-07 |
| L | H | 7 | 0.052698629 | 0.0143229267 | 0.0910743308 | 1.021982e-03 |
| L | P | 5.6 | -0.062840517 | -0.0981599009 | -0.0275211331 | 5.854899e-06 |
| L | P | 7 | -0.035467550 | -0.0717547674 | 0.0008196674 | 6.087072e-02 |
| P | A | 5.6 | 0.012884262 | -0.0173769659 | 0.0431454905 | 9.295196e-01 |
| P | A | 7 | 0.024500737 | -0.0049533801 | 0.0539548533 | 1.898278e-01 |
| P | C | 5.6 | -0.004233555 | -0.0352809203 | 0.0268138098 | 9.999883e-01 |
| P | C | 7 | 0.051857629 | 0.0224035127 | 0.0813117461 | 6.398452e-06 |
| P | H | 5.6 | 0.029413701 | -0.0008475275 | 0.0596749290 | 6.387608e-02 |
| P | H | 7 | 0.049181325 | 0.0197272083 | 0.0786354417 | 2.209409e-05 |
| P | L | 5.6 | 0.045236731 | 0.0149755023 | 0.0754979588 | 2.173489e-04 |
| P | L | 7 | 0.061651952 | 0.0321978350 | 0.0911060683 | 5.524740e-08 |

**Supplemental Table 8.** Linear model results showing the significance of fungal identity, competitor identity, and pH on performance in competition. An “s” in front of the genus represents the focal fungus, while “c” represents the competing fungus. Intercept uses *A. muscaria* and pH 5.6 as a baseline. Test was run under an assumption of alpha = 0.05.

|  | Estimate | | Std. Error | t value | Pr(>\|t\|) |
| --- | --- | --- | --- | --- | --- |
| Intercept | 1.214 | | 0.07772 | 15.62 | 2.445e-41******* |
| s.Cenococcum | 0.4529 | | 0.08974 | 5.047 | 7.526e-07******* |
| s.Hebeloma | -0.1333 | | 0.08974 | -1.485 | 0.1385****** |
| s.Laccaria | -0.5101 | | 0.06346 | -8.038 | 1.754e-14******* |
| s.Paxillus | -0.2628 | | 0.08974 | -2.928 | 0.003651******* |
| pH 7 | -0.04761 | | 0.1109 | -0.4293 | 0.668 |
| c.Cenococcum | -0.07497 | | 0.08974 | -0.8354 | 0.4041 |
| c.Hebeloma | -0.2085 | | 0.08974 | -2.323 | 0.02081****** |
| c.Laccaria | -0.3192 | | 0.08974 | -3.557 | 0.0004313******* |
| c.Paxillus | 0.007075 | | 0.06346 | 0.1115 | 0.9113 |
| s.Cenococcum : pH 7 | -0.2409 | | 0.1287 | -1.872 | 0.06206* |
| s.Hebeloma : pH 7 | -0.335 | | 0.1329 | -2.52 | 0.01222****** |
| s.Laccaria : pH 7 | 0.05037 | | 0.08974 | 0.5612 | 0.575 |
| s.Paxillus : pH 7 | 0.01446 | | 0.1278 | 0.1132 | 0.91 |
| s.Hebeloma : c.Cenococcum | -0.1263 | | 0.1122 | -1.126 | 0.2609 |
| s.Laccaria : c.Cenococcum | 0.08885 | | 0.1052 | 0.8443 | 0.3991 |
| s.Paxillus: c.Cenococcum | 0.1385 | | 0.1109 | 1.249 | 0.2127 |
| s.Cenococcum : c.Hebeloma | 0.1332 | | 0.1122 | 1.187 | 0.2361 |
| s.Laccaria : c.Hebeloma | 0.1727 | | 0.08974 | 1.925 | 0.05514* |
| s.Paxillus : c.Hebeloma | 0.1398 | | 0.1099 | 1.272 | 0.2044 |
| s.Cenococcum : c.Laccaria | 0.4191 | | 0.1229 | 3.41 | 0.0007319******* |
| s.Hebeloma : c.Laccaria | 0.2262 | | 0.1099 | 2.058 | 0.04042****** |
| s.Paxillus : c.Laccaria | 0.1848 | | 0.1099 | 1.681 | 0.09366***** |
| s.Cenococcum : c.Paxillus | -0.4072 | | 0.09098 | -4.475 | 1.059e-05******* |
| s.Hebeloma : c.Paxillus | -0.1373 | | 0.08974 | -1.53 | 0.1271 |
| pH 7 : c.Cenococcum | 0.2038 | | 0.1287 | 1.584 | 0.1142 |
| pH 7 : c.Hebeloma | 0.1128 | | 0.1329 | 0.8482 | 0.397 |
| pH 7 : c.Laccaria | 0.1418 | | 0.1287 | 1.102 | 0.2713 |
| pH 7 : c.Paxillus | 0.1396 | | 0.09098 | 1.535 | 0.1258 |
| s.Hebeloma : pH 7 : c.Cenococcum | -0.03134 | | 0.1633 | -0.1919 | 0.848 |
| s.Laccaria : pH 7 : c.Cenococcum | -0.2635 | | 0.1399 | -1.883 | 0.06053***** |
| s.Paxillus : pH 7 : c.Cenococcum | -0.3752 | | 0.1576 | -2.381 | 0.01786****** |
| s.Cenococcum : pH 7 : c.Hebeloma | -0.4185 | | 0.1633 | -2.562 | 0.01085****** |
| s.Laccaria : pH 7 : c.Hebeloma | -0.01483 | | 0.134 | -0.1107 | 0.912 |
| s.Paxillus : pH 7 : c.Hebeloma | -0.1414 | | 0.1604 | -0.8815 | 0.3787 |
| s.Cenococcum : pH 7 : c.Laccaria | -0.3798 | | 0.1676 | -2.266 | 0.02409****** |
| s.Hebeloma : pH 7 : c.Laccaria | -0.1256 | | 0.1626 | -0.7725 | 0.4404 |
| s.Paxillus : pH 7 : c.Laccaria | -0.1538 | | 0.1569 | -0.9806 | 0.3275 |
| s.Cenococcum : pH 7 : c.Paxillus | 0.1793 | | 0.1295 | 1.384 | 0.1672 |
| s.Hebeloma : pH 7 : c.Paxillus | -0.05569 | | 0.1329 | -0.4189 | 0.6755 |
| Adjusted R2  Residual Std. Error  F Statistic | | 0.782  0.142 (df = 322)  34.155*** (df = 39; 322) | | | |
| Note: *p<0.1; **p<0.05; ***p<0.01 | | | | | |

**Supplemental Table 9.** Linear model results showing the correlation between phylogenetic distance and growth rate distance. Intercept uses phylogenetic distance as a baseline. Test was run under an assumption of alpha = 0.05.

|  | Estimate | Std. Error | t value | Pr(>\|t\|) |
| --- | --- | --- | --- | --- |
| Intercept | 1.3501 | 0.0721 | 18.717 | 4.6e-05******* |
| Growth Rate Distance | -0.1969 | 0.7419 | -0.265 | 0.791 |
| Note: *p<0.1; **p<0.05; ***p<0.01 | | | | |

**Supplemental Table 10.** Anova results showing the comparison between phylogenetic distance and growth rate distance models run with four taxa (without *C. geophilum*).

|  | npar | AIC | BIC | logLik | deviance | Chisq | Df | Pr(>Chisq) |
| --- | --- | --- | --- | --- | --- | --- | --- | --- |
| Growth Rate Distance | 6 | -114.1290 | -93.6058 | 63.0645 | -126.1290 |  |  |  |
| Phylogenetic Distance | 7 | -101.88 | -77.931 | 57.937 | -115.88 | 0 | 1 | 1 |

**Supplemental Table 11.** Anova results showing the comparison between phylogenetic distance and growth rate distance models run with four taxa (without *C. geophilum* & *P. involutus*).

|  | npar | AIC | BIC | logLik | deviance | Chisq | Df | Pr(>Chisq) |
| --- | --- | --- | --- | --- | --- | --- | --- | --- |
| Growth Rate Distance | 6 | -16.4667 | -0.4861 | 14.2333 | -28.4667 |  |  |  |
| Phylogenetic Distance | 7 | -11.443 | 7.2015 | 12.721 | -25.442 | 0 | 1 | 1 |
